# Donor T cell DNMT3a regulates alloreactivity in mouse models of hematopoietic stem cell transplantation

**DOI:** 10.1172/JCI158047

**Published:** 2022-07-01

**Authors:** Yiouli P. Ktena, Michael A. Koldobskiy, Michael I. Barbato, Han-Hsuan Fu, Leo Luznik, Nicolas J. Llosa, Azeb Haile, Orly R. Klein, Chen Liu, Christopher J. Gamper, Kenneth R. Cooke

**Affiliations:** 1 Department of Oncology, Sidney Kimmel Comprehensive Cancer Center, Johns Hopkins School of Medicine, Baltimore, Maryland, USA.; 2 Department of Pathology, Yale School of Medicine, New Haven, Connecticut, USA.

**Keywords:** Transplantation, Bone marrow transplantation, Cellular immune response, Epigenetics

## Abstract

DNA methyltransferase 3a (DNMT3a) is an important part of the epigenetic machinery that stabilizes patterns of activated T cell responses. We hypothesized that donor T cell DNMT3a regulates alloreactivity after allogeneic blood and marrow transplantation (allo-BMT). T cell conditional *Dnmt3a* KO mice were used as donors in allo-BMT models. Mice receiving allo-BMT from KO donors developed severe acute graft-versus-host disease (aGVHD), with increases in inflammatory cytokine levels and organ histopathology scores. KO T cells migrated and proliferated in secondary lymphoid organs earlier and demonstrated an advantage in trafficking to the small intestine. Donor T cell subsets were purified after BMT for whole-genome bisulfite sequencing (WGBS) and RNA-Seq. KO T cells had global methylation similar to that of WT cells, with distinct, localized areas of hypomethylation. Using a highly sensitive computational method, we produced a comprehensive profile of the altered epigenome landscape. Hypomethylation corresponded with changes in gene expression in several pathways of T cell signaling and differentiation. Additionally, *Dnmt3a*-KO T cells resulted in superior graft-versus-tumor activity. Our findings demonstrate a critical role for DNMT3a in regulating T cell alloreactivity and reveal pathways that control T cell tolerance. These results also provide a platform for deciphering clinical data that associate donor DNMT3a mutations with increased GVHD, decreased relapse, and improved survival.

## Introduction

Allogeneic blood and marrow transplantation (allo-BMT) remains the only curative therapy for many malignant and nonmalignant disorders (1). Unfortunately, successful outcomes are limited by graft-versus-host disease (GVHD) and malignant relapse. Therapies that effectively mitigate GVHD without compromising graft-versus-tumor (GVT) activity remain elusive (2). Recent advances have improved knowledge of how the immune system discriminates between nonmalignant self and residual malignancy. However, a more complete understanding of the molecular underpinnings of alloreactivity following allo-BMT remains an unmet need toward improving patient outcomes (3).

The pathophysiology of acute GVHD (aGVHD) is complex and is hypothesized to comprise 3 distinct phases. Initially, the conditioning regimen causes diffuse damage and inflammation in host tissues. This proinflammatory environment facilitates activation of donor T cells by host antigen-presenting cells (APCs). The generation of cellular effectors in conjunction with the release of soluble inflammatory proteins culminates in damage of target tissues (4–6). Since its conception, this paradigm has been refined and challenged (4, 7–9). Yet the hypothesis still underscores the well-established fact that donor T cells are central participants in the immune dysregulation that characterizes GVHD, and it identifies opportunities to modulate this process. GVHD is closely linked to the GVT effect, the main effectors of which are cytotoxic donor T cells, which recognize allogeneic major or minor histocompatibility antigens and tumor neoantigens (4).

Epigenetic regulation is defined by posttranslational histone modification and methylation of cytosine residues of CpG dinucleotides within regulatory regions of genes (10). These changes, which control target gene transcription, are reproduced on nascent daughter DNA strands and are therefore somatically inheritable after cell division. Importantly, they can also be reversible during transitions between cellular fates. Hence, epigenetic regulation of gene expression has moved to the forefront of scientific interest. Epigenetic silencing is critical for normal terminal differentiation of tissues in the body and is frequently dysregulated in cancer (4). In recent years, it has become increasingly evident that T cell responses are heavily regulated by epigenetic mechanisms, including DNA methylation (11, 12).

In the clinical setting, allo-BMT recipients have been found to stably maintain the donor’s global methylation status, which is affected by evolution of donor chimerism (13). Moreover, methylation levels in critical genes such as *IFNG* and *FASL* have been shown to associate with the severity of aGVHD (13). Donor-specific epigenetic signatures that correlate with aGVHD severity have been proposed as a marker to be used alongside HLA typing to optimize donor selection (13). Importantly, recently published data revealed that patients with hematological malignancies receiving allo-BMT from healthy donors harboring incidental *DNMT3A* mutations (most commonly loss-of-function) associated with asymptomatic clonal hematopoiesis of indeterminate potential (CHIP) have higher rates of chronic GVHD and lower risk of malignant relapse (14). Subsequent data revealed that donor *DNMT3A* mutations were independently associated with improved overall survival and reduced risk of relapse, while other CHIP-associated mutations, such as *TET2*, were not (15). Of note, this effect was abrogated in patients receiving posttransplantation cyclophosphamide (PT-Cy) as a GVHD prophylaxis, suggesting that this phenomenon is at least partially mediated by effects on donor T cells (15).

We hypothesized that DNMT3a-dependent DNA methylation of donor T cells plays a critical role in the development of alloreactivity after BMT. We used mice with conditional *Dnmt3a* deletion in T cells as donors in well-established preclinical BMT models to test this hypothesis. We demonstrate that transplantation of *Dnmt3a*-KO T cells conferred significantly more rapid and severe GVHD, as well as improved GVT activity. We then applied a comprehensive genome-wide epigenomic landscape analysis following whole-genome bisulfite sequencing (WGBS), which to our knowledge has never been applied before to study GVHD. Finally, we performed RNA-Seq and gene set enrichment analysis (GSEA) to correlate the alterations in DNA methylation with changes in gene expression, highlighting pathways intimately involved in T cell activation and differentiation.

## Results

### Donors with conditional KO of Dnmt3a in T cells are immunophenotypically indistinguishable from WT littermate controls.

Germline *Dnmt3a* deletion results in death by 4 weeks of age; therefore, mice with conditional KO in T cells were generated as previously described (16–18). T cell–specific KO offspring are healthy, with normal body size (18). We collected spleen, thymus, lymph nodes, and bone marrow (BM) from KO mice and WT littermates. No differences between groups were seen with respect to cellularity, CD4^+^ and CD8^+^ distribution, or numbers of splenic Tregs (Supplemental Figure 1, A–C; supplemental material available online with this article; https://doi.org/10.1172/JCI158047DS1), in keeping with prior observations (18). In parallel experiments, T cells from KO and WT C57BL/6J (B6) animals were purified and stimulated in vitro with splenic dendritic cells (DCs) isolated from B6D2F1 (F1) mice. No differences in proliferation (Supplemental Figure 1D), cytolytic activity (Supplemental Figure 1E), or cytokine production (data not shown) were observed between groups.

### Loss of donor T cell Dnmt3a results in accelerated severe aGVHD.

**Figure 1:**
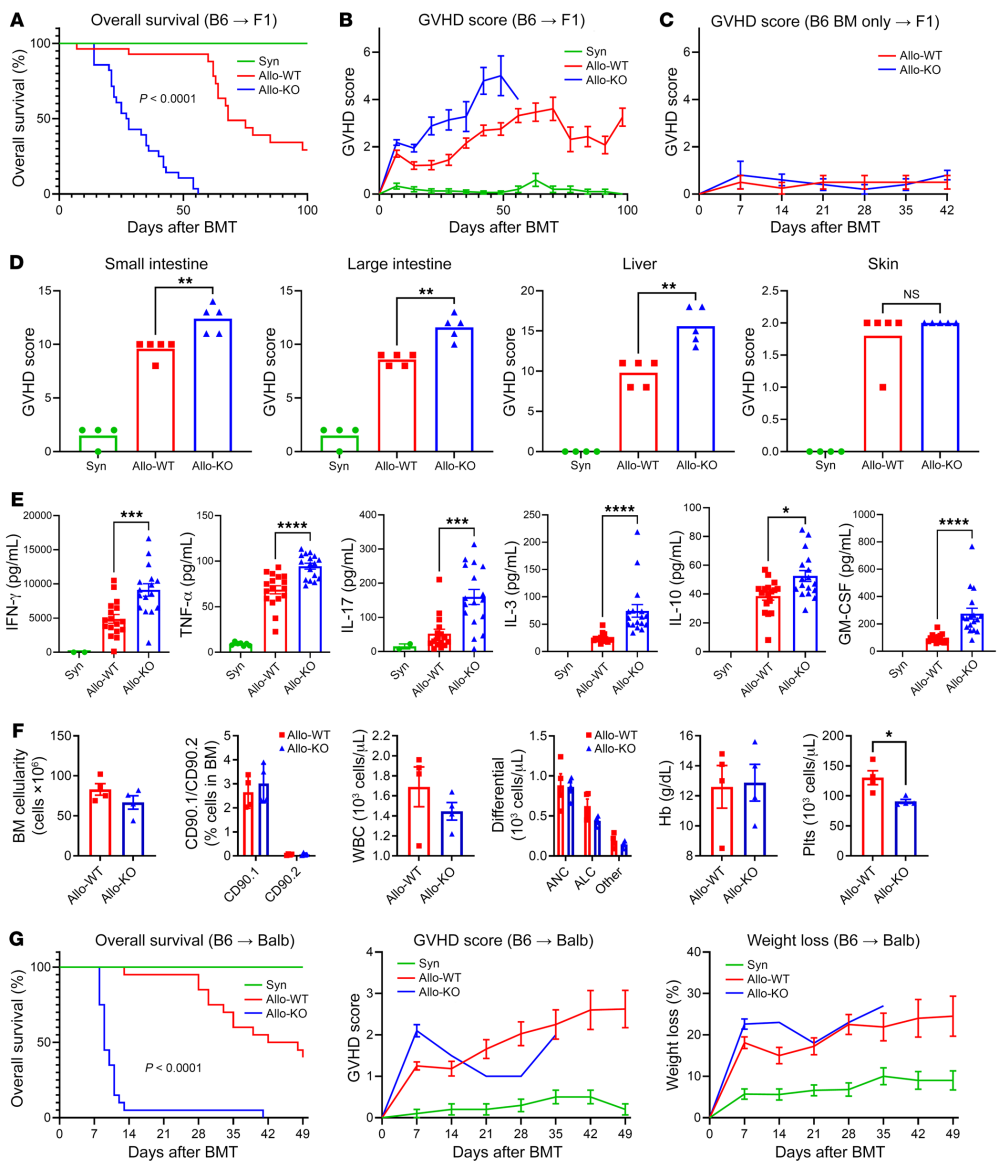
DNMT3a-deficient T cells result in accelerated experimental aGVHD. Lethally irradiated recipients received BMT from syngeneic (Syn), allogeneic WT (Allo-WT), or *Dnmt3a*-KO donors (Allo-KO). Haploidentical B6→F1 model: (**A**) overall survival, (**B**) clinical GVHD score, (**C**) survival in the absence of T cells and with T cell–depleted BM only. Data from 3 experiments; Syn *n =* 15, Allo *n =* 28 each. (**D**) Histopathological organ-specific GVHD scores on day +7; *n =* 4–5 per group. (**E**) Serum cytokine levels by multiplex bead assay on day +7. Data from 3 experiments; Syn *n =* 8, Allo *n =* 17 each. (**F**) BM cellularity, T cell chimerism, and peripheral blood counts on day +14; *n =* 4–5 per group. (**G**) Fully mismatched B6→BALB/cJ model. Left to right: Survival, clinical GVHD score, weight loss. Data from 2 experiments; Syn *n =* 10, Allo *n =* 20 each. *P < 0.05, **P < 0.01, ***P < 0.001, ****P < 0.0001, Mann-Whitney *U* test, Mantel-Cox log-rank test for survival data. ALC, absolute lymphocyte count; ANC, absolute neutrophil count; Hb, hemoglobin; Plt, platelets.

To define the effect of *Dnmt3a* gene deletion in donor T cells on alloreactivity, we used an established haploidentical model wherein B6 (H-2^b^) and F1 (H-2^bxd^) mice serve as BMT donors and recipients, respectively, as described in Methods. Recipients of B6 *Dnmt3a*-KO donors exhibited more severe, systemic aGVHD, as measured by survival and clinical score, when compared with mice receiving allo-BMT from WT donors (Figure 1, A and B). As expected, syngeneic BMT recipients did not develop GVHD and were ultimately indistinguishable from nontransplanted controls. Similarly, mice receiving T cell–depleted WT or KO BM only did not develop GVHD (Figure 1C). Severe, systemic GVHD observed following BMT with *Dnmt3a*-KO donors was associated with increased histopathology scores in the gut (small and large intestine) and liver, but not skin, compared with allogeneic controls (Figure 1D). Proinflammatory cytokines, including IFN-γ, TNF-α, GM-CSF, IL-17, IL-3, and IL-10, were found to be significantly increased in the serum of KO recipients on day 7 after BMT (day +7) by Luminex multiplex immunoassay (Figure 1E) (19, 20). To rule out graft failure as a contributor to early mortality, as was seen by others when pharmaceutical hypomethylating agents were administered early after BMT (21), we assessed marrow cellularity and T cell chimerism along with peripheral blood components on day +7. KO recipients exhibited robust BM engraftment and donor-derived hematopoiesis (Figure 1F); peripheral WBC, differential, and hemoglobin levels were comparable among the groups, whereas as platelet recovery, while still adequate, was lower in KO recipients, likely reflective of severe systemic GVHD (22). To ensure our observations were not due to a strain-dependent phenomenon, we conducted similar BMT experiments in a widely used MHC-disparate system (B6→BALB/cJ). As shown in Figure 1G, GVHD severity was again noted to be significantly increased with DNMT3a-KO compared with WT control donors.

### Accelerated aGVHD observed in the absence of donor T cell Dnmt3a gene expression is driven primarily by CD8^+^ cells.

**Figure 2:**
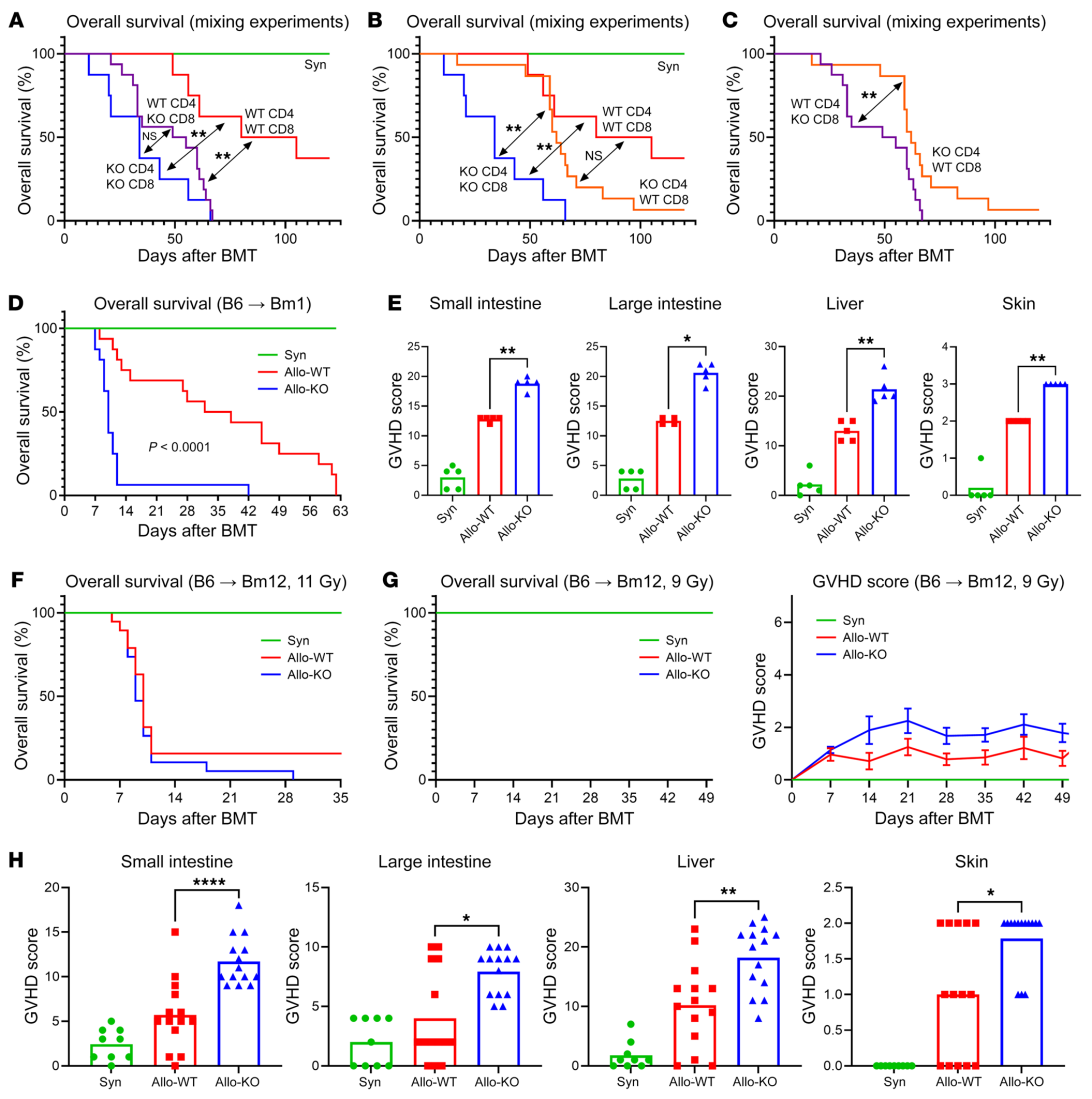
DNMT3a loss in CD8^+^ T cells is sufficient to result in increased GVHD. (**A**–**C**) Survival in mixing experiments wherein CD4^+^ and CD8^+^ WT and KO T cells were separately isolated and coinjected at a CD4/CD8 ratio of 2:1 in various combinations. Data from 2 experiments; Syn, WT, KO *n =* 8 each, mixed groups *n =* 16 each. (**D**) Survival in the B6→Bm1 model in which donor and recipient differ only in MHC I and graft contains solely CD8^+^ T cells. Data from 2 experiments; Syn *n =* 9, Allo *n =* 16 each. (**E**) Histopathological organ-specific GVHD scores in the B6→Bm1 model on day +7; *n =* 5 per group. (**F**) B6→Bm12 model in which donor and recipient differ only in MHC II and graft contains solely CD4^+^ T cells: survival at a TBI dose of 11 Gy. Data from 2 experiments, syn *n =* 10, allo *n =* 19 each. (**G**) B6→Bm12 with a TBI dose of 9 Gy: survival and clinical GVHD score. Note that all three groups maintained 100% survival. (**H**) Histopathological GVHD scores. Data from 2 experiments; Syn *n =* 9, Allo *n =* 14 each. *P < 0.05, **P < 0.01, ****P < 0.0001, Mann-Whitney *U* test, Mantel-Cox log-rank test for survival.

CD4^+^ and CD8^+^ T cells both contribute to GVHD in the haploidentical model described above (23–27). To more carefully ascertain the contribution of each T cell subset, we performed mixing experiments wherein WT and KO CD4^+^ and CD8^+^ T cells were separately isolated and coinjected at a CD4/CD8 2:1 ratio in different combinations. The number of experimental groups was expanded to include those that received WT CD4^+^ T cells with KO CD8^+^ T cells, and vice versa. All mice receiving CD8^+^ *Dnmt3a*-deficient T cells experienced severe GVHD, with early death regardless of the genotype of the CD4^+^ cells (Figure 2A). The contribution of KO CD4^+^ T cells alone to GVHD severity was less impactful; death before day +50 was rare, and survival was not significantly different from that of recipients of WT cells (Figure 2B). Moreover, recipients of KO CD4^+^ and WT CD8^+^ T cells demonstrated superior survival compared with animals receiving KO CD4^+^ and KO CD8^+^ T cells (Figure 2C). To extend these observations, we used additional GVHD models (Supplemental Table 1) wherein donors and recipients differ solely in MHC class I (MCH-I): B6 (H-2^b^) donors into B6.C-H2^bm1^/ByJ H-2^b^ (Bm1; H-2^b^) recipients; or MCH-II: B6 (H-2^b^) donors into B6.C-H2^bm12^/KhEgJ (Bm12; H-2^b^) recipients. In these models, the graft contained, respectively, only purified CD8 or CD4. Therefore, the development of GVHD is dependent solely on either CD8 (MHC-I–mismatched model) or CD4 (MHC-II–mismatched model). The severity of GVHD was even more pronounced in the MHC-I–based model following *Dnmt3a*-KO BMT (Figure 2D), where it was again associated with marked changes in histopathology scores in all target organs (Figure 2E). In the MHC-II–mismatched model, WT and KO recipients developed severe GVHD following BMT with 11 Gy total body irradiation (TBI), consistent with the robust early cytokine release characteristic of this model (28–30); however, no differences between groups were noted (Figure 2F). The TBI dose was subsequently reduced to 9 Gy in order to mitigate early mortality and provide the opportunity to study the effect of DNMT3a activity in an established model of chronic GVHD; as previously described, reducing the TBI dose in certain strain combinations can result in a chronic GVHD phenotype by modifying the synergistic effect of TBI and T cell dose on induction of alloreactivity (29, 31, 32). In this context, systemic GVHD as assessed by survival and clinical score was mild and similar in WT and KO recipients (Figure 2G). However, mice receiving BMT from *Dnmt3a*-KO donors had more severe target organ GVHD by the end of the observation period, on day +50 (Figure 2H).

### A CD4^+^CD8^+^ double-positive T cell population emerges in Dnmt3a-KO T cell recipients.

**Figure 3:**
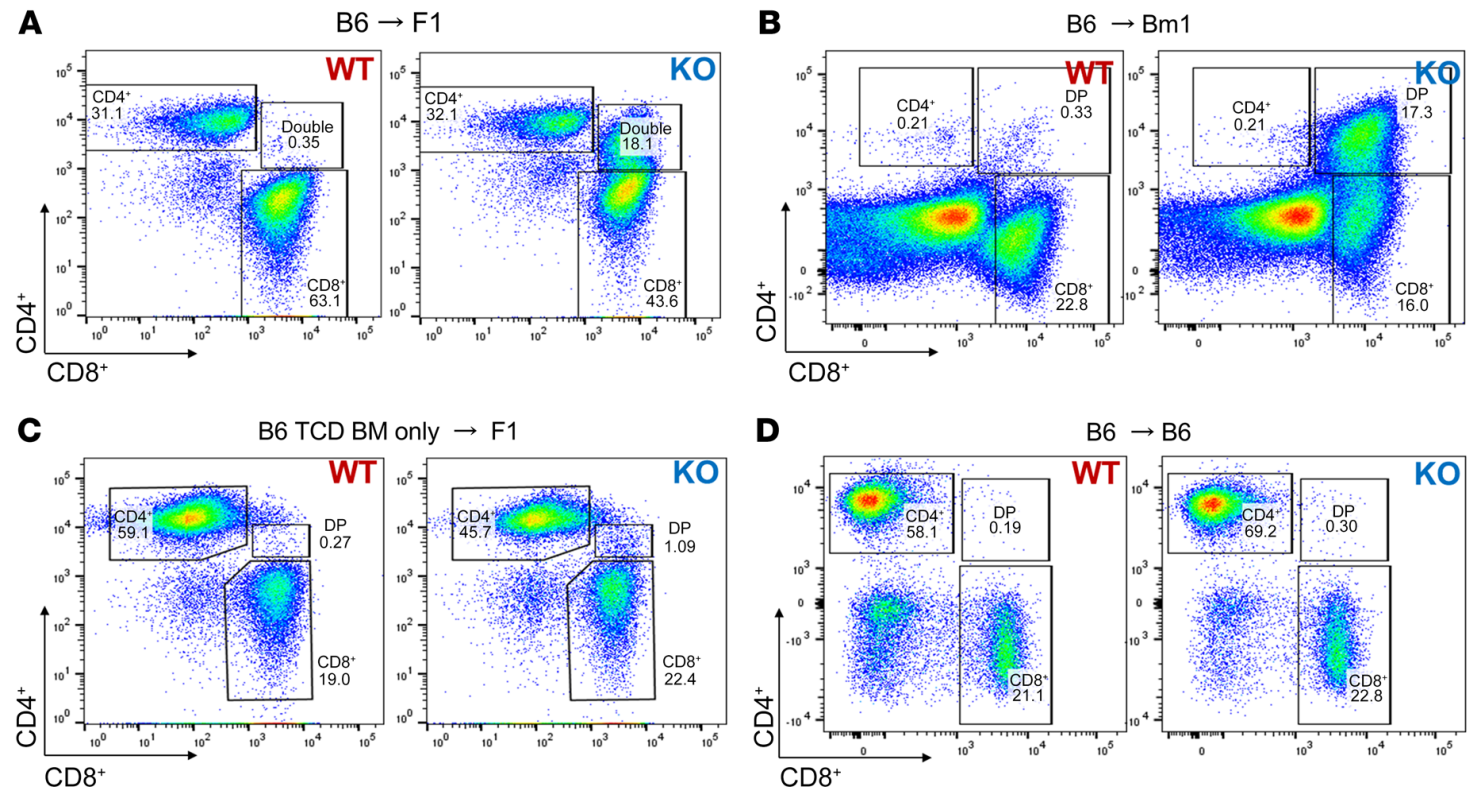
Increased GVHD correlates with the emergence of a distinct CD4^+^CD8^+^ T cell population in recipients of *Dnmt3a*-KO T cells. Flow cytometry of CD4 versus CD8 on splenic T cells, showing the marked expansion of the DP population on day +7 in the B6→F1 (**A**) and B6→Bm1 (**B**) models, but not in a B6→F1 model with BM only (**C**) or a syngeneic B6→B6 model with BMT from either WT or KO donors (**D**). TCD, T cell–depleted.

In parallel experiments using the B6→F1 system, mice were sacrificed on days +7 and +14. Analysis of single-cell suspensions of spleen and lymph nodes by multicolor flow cytometry identified a distinct CD4^+^CD8^+^ double-positive (DP) population of T cells that was dramatically expanded in recipients of *Dnmt3a*-KO T cells (Figure 3A). This expanded population is not present in naive *Dnmt3a*-KO donors at baseline (18). The morphology revealed by the flow cytometry plots suggests that this population emerged from the CD8^+^ subset, and the cytokine expression profile of this cell population most closely resembled that of CD8^+^ T cells (Supplemental Figure 3). Indeed, in the CD8-dependent MHC-I model presented above (Figure 2D), this population was also present (Figure 3B). By conducting experiments using only allogeneic T cell–depleted WT or KO BM without T cells, we showed that the DP population emerged from expansion of a naive, alloreactive, splenic T cell population rather than from de novo aberrant thymopoiesis (Figure 3C). The DP population was also absent in the context of homeostatic T cell expansion in recipients of syngeneic BMT from either WT or KO donors (Figure 3D).

### Disruption of Dnmt3a gene expression enhances early T cell proliferation and migration to secondary lymphoid organs and the gastrointestinal tract after allo-BMT.

**Figure 4:**
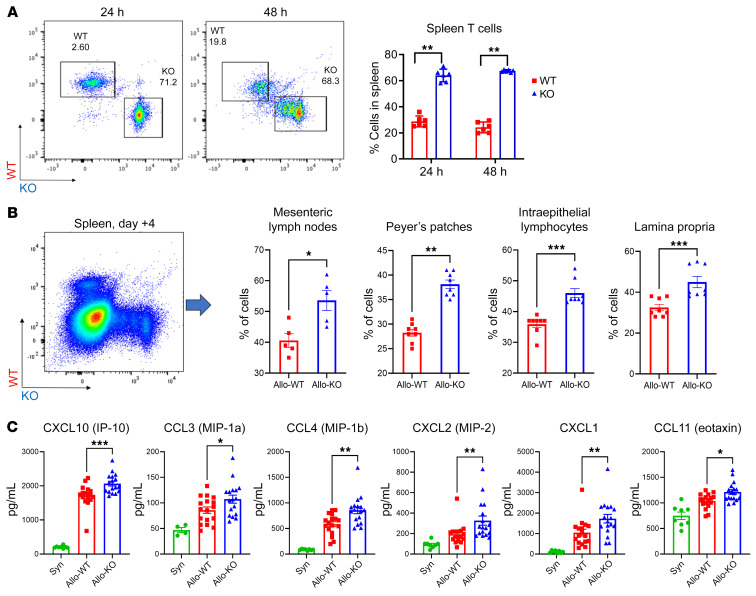
Loss of DNMT3a expression results in a trafficking advantage for donor T cells to SLOs. Purified WT and KO B6 T cells were stained with CSFE and e450, respectively (and vice versa in replicate experiments) and were coadoptively transferred at a 1:1 ratio (3 × 10^6^ to 5 × 10^6^ cells each) into lethally irradiated allogeneic F1 animals. Spleen (**A**) and lymph node (not shown) flow cytometry was performed 24 and 48 hours later. Data from 2 experiments; *n =* 6 per time point per group. (**B**) On day +4, most cells lost the proliferation dye. WT and KO populations were distinguished via allelic differences between CD45.1/2 and CD90.1/2. Data from 2 experiments; *n =* 8 per group (except MLNs *n =* 5). (**C**) Serum chemokine levels by multiplex bead assay in the B6→F1 model on day +7. Data from 3 experiments; Syn *n =* 8, Allo *n =* 17 each. *P < 0.05, **P < 0.01, ***P < 0.001, Mann-Whitney *U* test or Mantel-Cox log-rank test for survival data.

To investigate the cause of the enhanced GVHD severity observed in mice receiving *Dnmt3a*-KO BMT, we next examined whether *Dnmt3a* gene deletion influenced T cell migration to secondary lymphoid organs (SLOs) early after infusion. Purified B6 WT and *Dnmt3a*-KO T cells were differentially stained with fluorescent proliferation dyes (described in Methods) and adoptively cotransferred at a dose of 3 × 10^6^ to 5 × 10^6^ cells into lethally irradiated F1 mice at a 1:1 ratio. Mice were examined at 24 and 48 hours after T cell transfer, and flow cytometry was performed on SLOs. A significant increase in the percentage of *Dnmt3a*-KO cells was noted in the spleen at 24 and 48 hours, demonstrating that *Dnmt3a*-KO T cells migrated to SLOs more efficiently and robustly than WT cells. The more precipitous loss of vital dye supports an early proliferation advantage in the KO T cells (Figure 4A). Additionally, lower expression of caspase-3/7 at the 48-hour time point in the KO T cells indicated potentially reduced apoptosis in the absence of DNMT3a (Supplemental Figure 4). On day +4, a large portion of cells had lost the proliferation dye, but WT and KO T cells were tracked and differentiated by flow based on allelic differences in CD45 and CD90. At this early time point, KO T cells were also found at higher percentages in mesenteric lymph nodes (MLNs) and Peyer’s patches (PPs), as well as in intraepithelial lymphocytes (IELs) and lamina propria (LP) of the gastrointestinal tract (Figure 4B). Interestingly, several chemokines that we and others have shown to be critical to effector cell migration in GVHD — including CXCL10 via CXCR3 (33, 34), CCL3 via CCR1 (35, 36), and CCL2 via CCR2 (37, 38) — were found to be significantly elevated in the serum of KO recipients on day +7 (Figure 4C). Next, we determined the effect of *Dnmt3a* gene deletion on splenic T cell expansion on days +7 and +14 in the B6→F1 model. Surprisingly, the absolute numbers of CD4^+^ and CD8^+^ T cells were not different between allogeneic groups. Further evaluation found no differences in the number or percentage of T cell memory subsets (by CD44 and CD62L expression) or Tregs (data not shown).

### Loss of Dnmt3a results in distinct areas of localized genomic hypomethylation.

**Figure 5:**
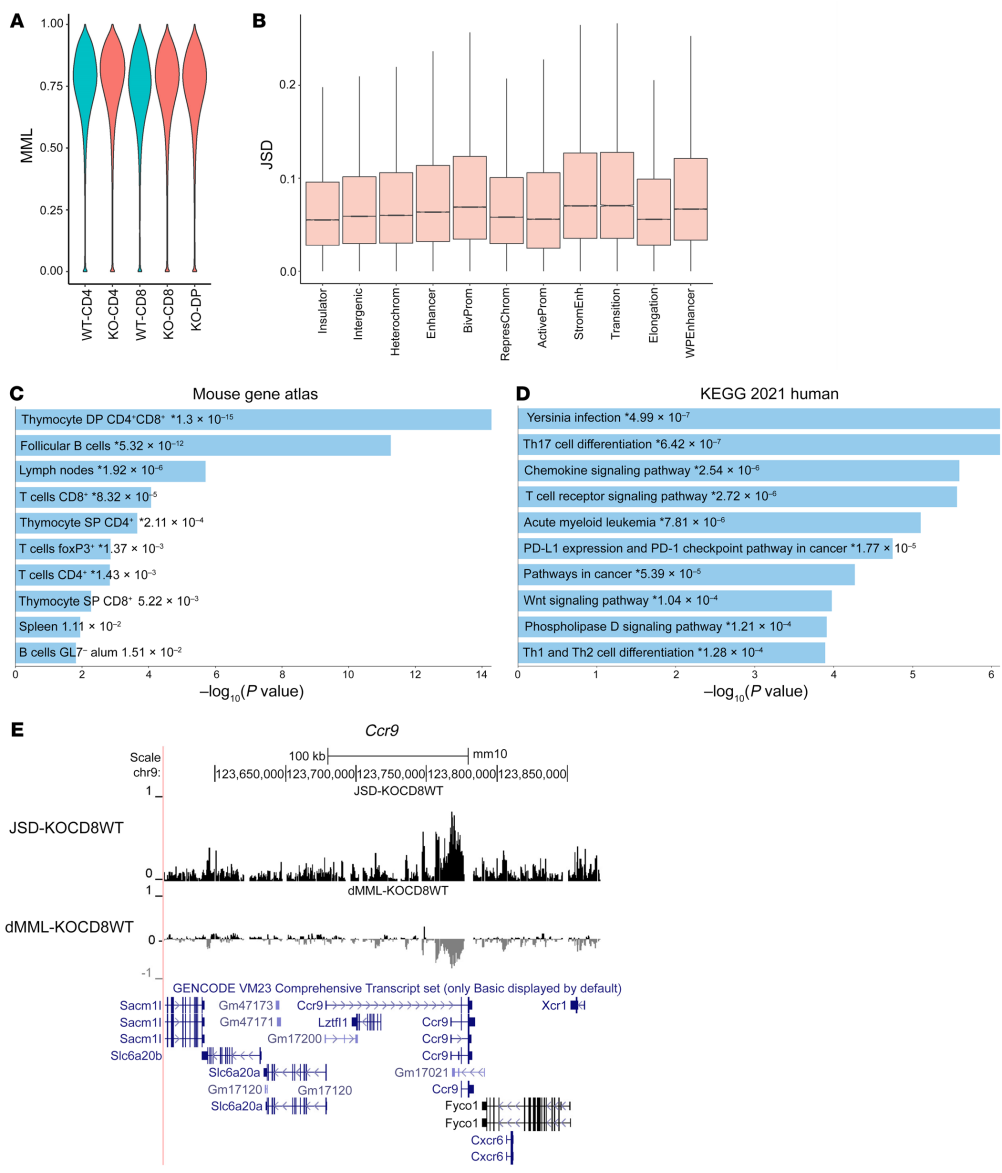
Loss of DNMT3a results in distinct areas of localized genomic hypomethylation. Splenic WT and KO CD4^+^, CD8^+^, and CD4^+^CD8^+^ T cells were isolated by flow cytometry on day +10 in the B6→F1 model, and underwent DNA and RNA extraction for WGBS and RNA-Seq. (**A**) Similar distribution of MMLs across all purified subsets. (**B**) Box plots of the JSD showing the comparison of KO and WT CD8^+^ T cell genomic features annotated by chromatin state and gene-regulatory function as an example. The JSD captures methylation discordance, whether due to dMMLs, methylation entropy, or other statistical factors (66). Differences localize over enhancer elements and promoters bearing bivalent marks. EnrichR analysis (90) of pathways enriched in the genes differentially methylated between experimental groups using the Mouse Gene Atlas (**C**) and KEGG 2021 Human databases (**D**). SP, single-positive. (**E**) The *Ccr9* gene promoter as an example of a top-ranked differentially methylated region between DNMT3a WT and KO CD8^+^ T cells. The peak in JSD indicates differential methylation, and the negative peak in dMML indicates hypomethylation in the KO cells.

To elucidate the DNMT3a-dependent changes in DNA methylation and gene expression underlying the inflammatory phenotype seen after *Dnmt3a*-KO BMT, we sought to comprehensively characterize DNA methylation and gene expression in relevant T cell subsets. Using the B6→F1 model described above, we isolated WT and KO CD4^+^, CD8^+^, and CD4^+^CD8^+^ splenic T cells on day +10 using flow cytometric sorting. Genomic DNA and RNA were isolated for WGBS and RNA-Seq, respectively. WGBS serves as the gold standard in DNA methylation analysis, providing single-base resolution assessment of DNA methylation at nearly all CpG sites in the genome (39). Importantly, lack of DNMT3a did not result in genome-wide hypomethylation in CD4^+^, CD8^+^, or CD4^+^CD8^+^ DP T cells; genome-wide distribution of DNA methylation levels was maintained between WT and KO T cells (Figure 5A). Rather, *Dnmt3a*-KO T cells exhibited focal hypomethylation over specific regulatory regions and genes. By evaluating DNA methylation alterations across genomic features annotated by their chromatin state and gene-regulatory function, we found that *Dnmt3a*-KO CD4^+^ and CD8^+^ T cells exhibited substantial hypomethylation of enhancer elements and promoters bearing bivalent marks (Figure 5B). We analyzed WT and KO T cells for differences in methylation over gene promoters, where DNMT3a-dependent DNA methylation has an important role in regulating gene expression (40). We ranked genomic promoter regions by Jensen-Shannon distance (JSD) of the DNA methylation probability distributions, which revealed differential methylation in WT and KO T cells (Supplemental Tables 2 and 3). The JSD captures methylation discordance, whether due to dMMLs, methylation entropy (stochasticity), or other statistical factors. Importantly, GSEA of promoters differentially methylated in WT and *Dnmt3a*-KO T cells also identified significant enrichment of gene sets related to T cell development and signaling (Figure 5, C and D), suggesting that focal DNMT3a-dependent DNA methylation alterations occur in functionally relevant gene targets affecting T cell function. One example of a top-ranked region differentially methylated between WT and *Dnmt3a-*KO CD8^+^ T cells is the *Ccr9* gene, which exhibited profound hypomethylation of its promoter in *Dnmt3a*-KO compared with WT cells (Figure 5E). CCR9 is known to be involved in GVHD pathogenesis and specifically contributes to T cell homing to intestinal tissues (41).

### Areas of differential methylation correspond to changes in gene transcription.

**Figure 6:**
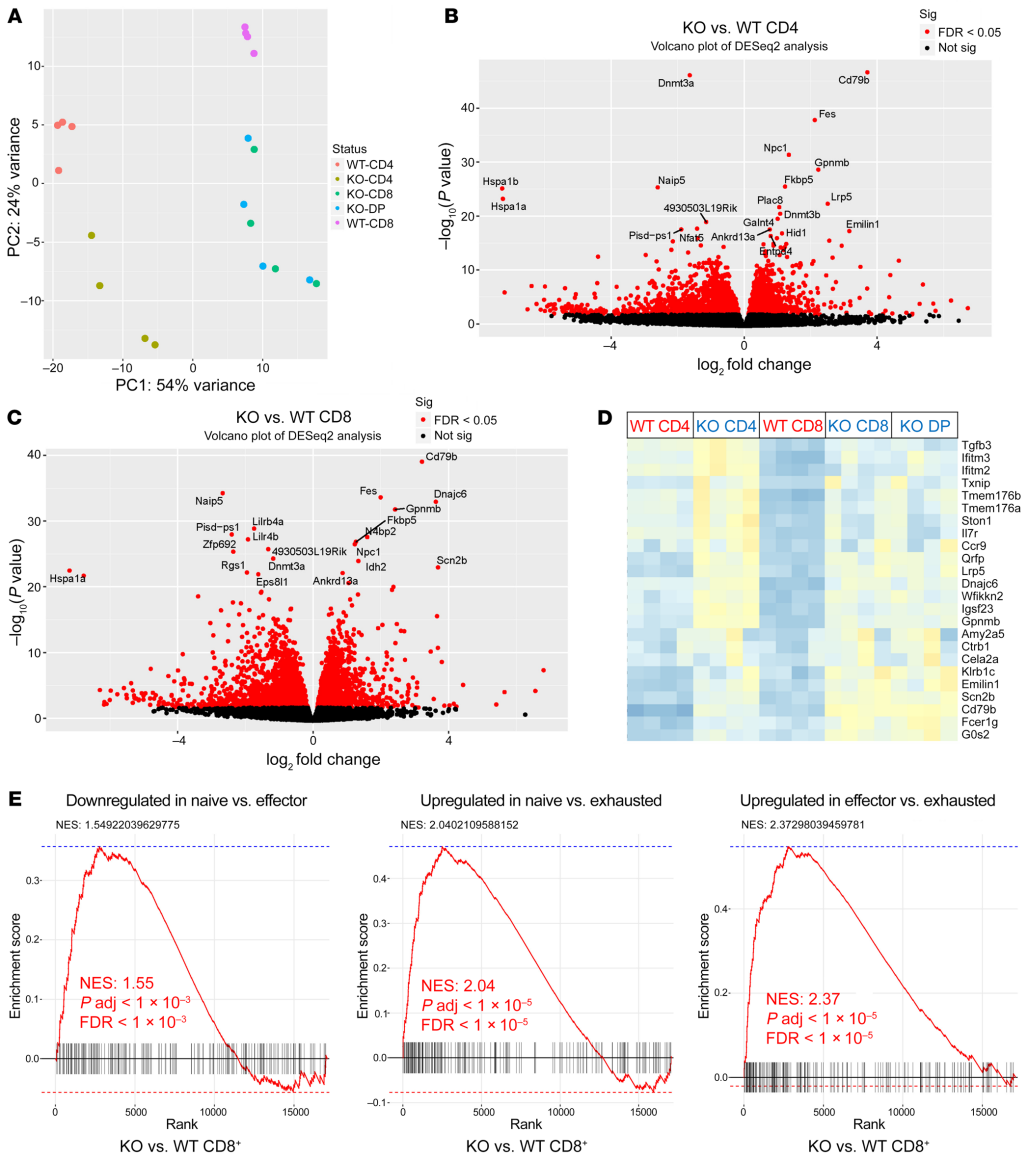
Areas of differential methylation correlate with changes in gene transcription. (**A**) PCA of whole-genome transcriptomes from RNA-Seq samples; *n =* 4 per group. (**B**) Volcano plot (log_10_ *P* values vs. log_2_ fold changes in expression) of differentially expressed transcripts in KO vs. WT CD4^+^ T cells and (**C**) in KO vs. WT CD8^+^ cells. sig, significance. (**D**) Heatmap cluster of top-ranked differentially expressed genes between T cell subsets. Each column represents a biological replicate. (**E**) Representative GSEA enrichment plots of selected immune-related gene sets overrepresented in KO vs. WT CD8^+^ T cells derived from the MSigDB C7 database. adj., adjusted.

Given the effect of DNA methylation on gene expression, we next assessed gene expression changes in *Dnmt3a*-KO versus WT T cells from the same data set. We performed principal component analysis (PCA) of gene expression by WT and KO CD4^+^, CD8^+^, and CD4^+^CD8^+^ DP cells. The data showed that Dnmt3a KO resulted in distinct gene expression profiles in CD4^+^ and CD8^+^ cells relative to their WT counterparts (Figure 6A). *Dnmt3a*-KO CD4^+^CD8^+^ DP cells demonstrated a gene expression profile similar to that of KO CD8^+^ T cells (Figure 6A). Differential gene expression analysis between WT and KO CD4^+^ T cells (Figure 6B and Supplemental Table 3) and WT and KO CD8^+^ T cells (Figure 6C and Supplemental Tables 4 and 5) identified both up- and downregulation of genes consequent to *Dnmt3a* loss. Interestingly, genes exhibiting upregulation in *Dnmt3a*-KO T cells (Figure 6D) included, among others, *Ccr*9, which is mentioned above; *Il7r*, whose expression has been associated with maintenance of Th1 effector cells in chronic infection (42); *Tmem176a* and *Tmem176b*, which have been shown to be expressed in Th17 cells (43); and IFN-γ–inducible molecules such as *Ifitm3* and *Ifitm2* (44). Using published gene sets from the Molecular Signature Database (MSigDB; C7, immunologic signature database), we conducted GSEAs of the differentially expressed genes in our data set and found significant overlap with data from previous studies of transcriptional programs involving regulation of T cell responses in various models of chronic infection, T cell effector differentiation, and T cell anergy or tolerance (45–49). We found that KO CD8^+^ T cells were highly enriched for effector-like signatures and negatively enriched for exhaustion-like signatures, while CD4^+^ T cells were enriched for genes expressed in activated and progenitor cell populations (Figure 6E and Supplemental Tables 6 and 7).

### Alterations in methylome and transcriptome provide mechanistic clues to the enhanced GVHD.

**Figure 7:**
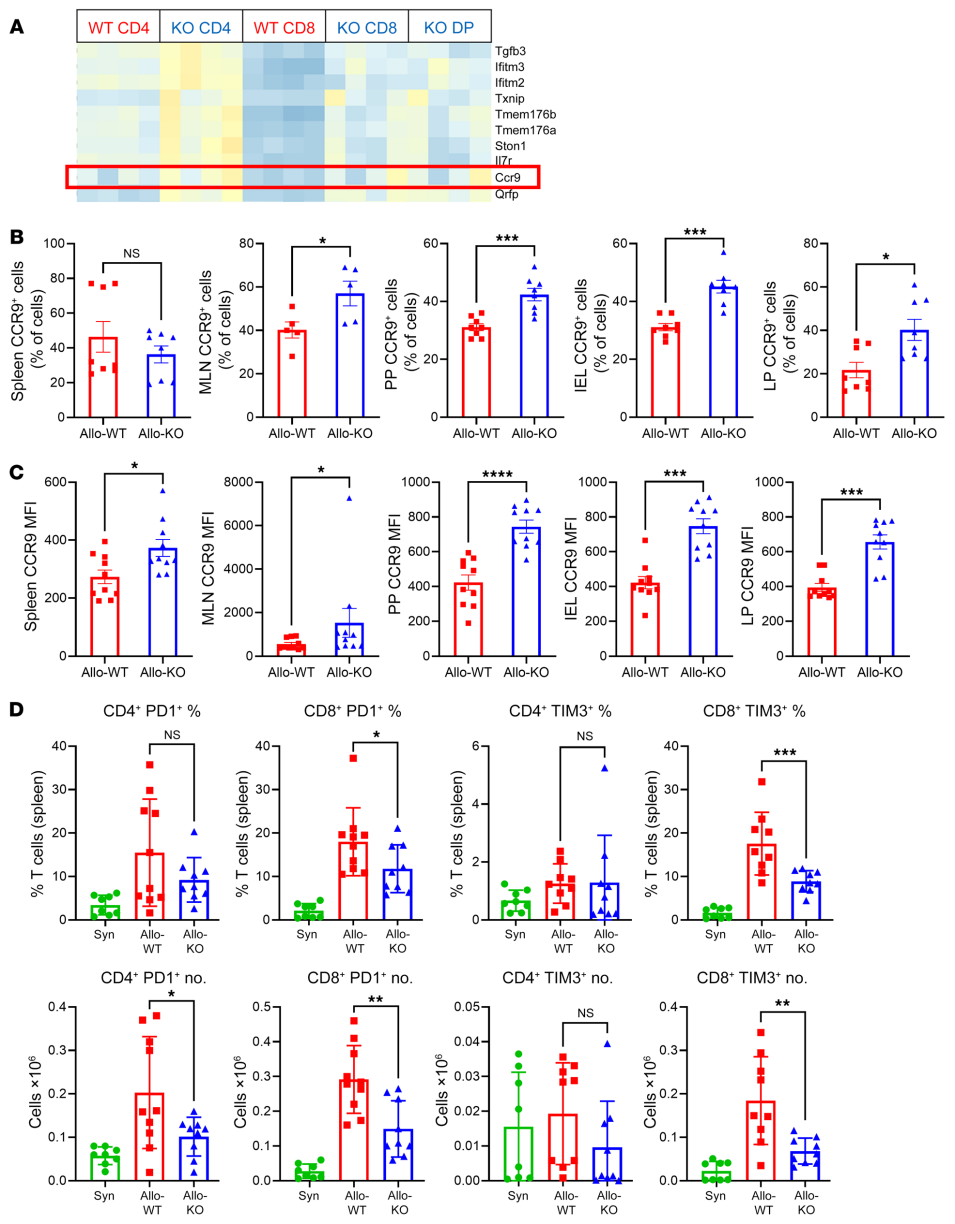
Alterations in methylome and transcriptome provide mechanistic clues for enhanced GVHD severity. (**A**) Close-up view of the heatmap presented in Figure 6D. (**B**) CCR9 expression by flow cytometry in the migration experiments presented in Figure 4B. On day +4, most of the CCR9^+^ cells in the spleen are WT, while the cells that have started migrating toward the MLNs, PPs, IELs, and LP are mostly KO. Data from 2 experiments; *n =* 8 per group (except MLNs *n =* 5). (**C**) MFI of CCR9. (**D**) PD-1 and TIM3 expression on CD4^+^ and CD8^+^ cells on day +7 in the B6→F1 model (top row: percentage of splenic T cells; bottom row: absolute numbers). No differences were detected in LAG3 expression. Data from 2 different experiments; Syn *n =* 8, Allo *n =* 9 per group. *P < 0.05, **P < 0.01, ***P < 0.001, ****P < 0.0001, Mann-Whitney *U* test or Mantel-Cox log-rank test for survival data.

As shown in Figure 5C, the *Ccr9* gene locus is hypomethylated in KO T cells after BMT. Additionally, *Ccr9* gene expression was significantly higher in *Dnmt3a*-deficient CD4^+^ and CD8^+^ T cells (Figure 7A and Table 1). CCR9 is a chemokine receptor critically involved in T cell trafficking to the small intestine (37). Its expression is highest in IELs and LP of the gastrointestinal tract; however, functional CCR9 expression has also been confirmed in MLNs and PPs, where gut-homing T cells are primed (50–53). In addition, CCR9:CCL25 receptor:ligand interactions have been implicated in GVHD pathophysiology (37, 54, 55). In the coadoptive transfer experiments as described above, on day +4, we found that a higher percentage of KO cells that had migrated to the MLNs, PPs, IELs, and LP expressed CCR9 than WT control cells (Figure 7B). CCR9 MFI was higher in the KO T cells across all compartments (Figure 7C).

To further investigate the GSEA results shown in Figure 6 and Supplemental Tables 6 and 7 — which indicated enrichment for genes involved in chronic infection responses (in agreement with previously published data showing a role for DNMT3a in effector fate decisions; ref. 16) — we examined memory T cell subsets in our BMT model and found no difference between the groups (data not shown). *Tcf7*, which has been associated with effector cell differentiation (16), was hypomethylated in the KO T cells but not differentially expressed (data not shown).

In a separate set of experiments, we assessed T cell exhaustion pathways. Transcriptional factors such as *Nfat5* and *Tox*, known to induce T cell hyporesponsiveness (56), were found to have significantly lower expression levels in splenic *Dnmt3a*-KO T cells after BMT in our RNA-Seq data (Table 2). Inhibitory markers such as PD-1 and TIM3, directly regulated by *Nfat* family genes and *Tox* (56), were underexpressed in the KO recipients (albeit with an adjusted *P* > 0.05); and indeed, lower expression in the CD8^+^ compartment was confirmed by flow cytometry (Figure 7D). Accordingly, targets such as *Pten* and *Pik3r1* that are downstream of and downregulated by PD-1 (57) were found to be overexpressed in the allo-KO recipients (Table 2). In our WGBS data, the loci of these genes and their promoters showed only mild differences in methylation, indicating that de novo DNA methylation affects numerous transcriptional pathways that control the development of T cell tolerance both directly and indirectly (Supplemental Figure 2).

### Dnmt3a-KO T cells convey superior GVT effects.

**Figure 8:**
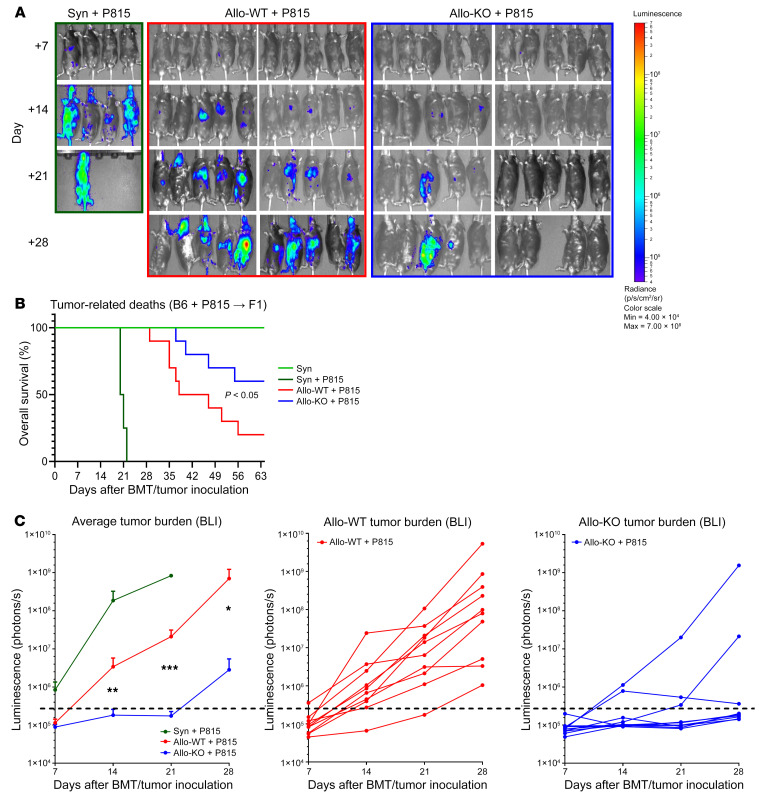
DNMT3a-KO T cells convey superior GVT effects. P815 tumor cells (500 cells per animal) were added to the inoculum on day 0 using the B6→F1 model. Tumor burden was monitored by BLI as described in Methods. (**A**) BLI images. (**B**) Tumor-related deaths (**C**) Average and individual tumor burden represented as photons per second. Data show 1 of 2 replicate experiments; Syn *n =* 5, Allo *n =* 10 per group per experiment. *P < 0.05, **P < 0.01, ***P < 0.001, Mann-Whitney *U* test or Mantel-Cox log-rank test for survival data. Radiance, p/s/cm^2^/sr. Color scale: min, 4.00 × 10^4^; max, 7.00 × 10^8^.

Given the observed effect that lack of donor T cell DNMT3a activity had on allogeneic responses after BMT, it was critical to define how loss of *Dnmt3a* gene expression would affect GVT activity. We did so using the haploidentical murine GVHD model represented in Figure 1. In these experiments, the splenic T cell dose was decreased by 50% in both allogeneic groups to minimize early GVHD-associated deaths, and a dose of 500 luciferase-expressing P815 tumor cells (H-2^d^) was added to the BM inoculum on day 0. BMT recipients were monitored daily for survival and weekly by bioluminescence. As expected, all syngeneic recipients receiving P815 cells died of widely disseminated tumor cell infiltration by day 25. By contrast allo-BMT using *Dnmt3a* WT donors resulted in evident GVT activity in the context of GVHD. Notably, recipients of *Dnmt3a*-KO T cells exhibited superior tumor control and/or eradication as compared with recipients of WT T cells, and this potent antitumor response was associated with improved tumor-free survival (Figure 8).

## Discussion

GVHD and suboptimal GVT activity remain 2 major contributors to morbidity and mortality after allogeneic BMT (58, 59). Expanding the current knowledge on the pathophysiology of these tightly intertwined processes is crucial for improving patient outcomes. Both GVHD and GVT responses fundamentally depend on donor T cell activation by host APCs. Furthermore, epigenetic modifications, such as de novo methylation as catalyzed by DNMT3a, critically influence T cell differentiation and plasticity restriction (16–18).

*Dnmt3a* is selectively upregulated 38-fold following TCR stimulation (18) and subsequently regulates Th cell polarization as well as effector T cell differentiation (16, 17). Specifically, data from our and other groups have shown that T cell effector function is epigenetically regulated to restrict patterns of effector gene expression dependent on the context of T cell activation (11, 16–18, 60). These patterns are propagated following cell division to daughter cells that participate in immunologic memory. For example, CD4^+^ Th1 cells express high levels of IFN-γ from an unmethylated locus, while *Il4* and *Foxp3* are silenced by DNA methylation (17). In contrast, Th2 cells demethylate and express high levels of IL-4, while *Ifng* and *Foxp3* are silenced by DNA methylation (17). Finally, Tregs express high levels of the transcription factor Foxp3, which controls their repressive function, while expression of *Ifng* and *Il4* is silenced by DNA methylation. Our group has also shown that T cell–specific deletion of *Dnmt3a* enhances the plasticity of Th cells and allows them to acquire alternative Th fates by reprogramming cytokine expression (17). In addition, *Dnmt3a*-KO CD8^+^ T cells can adopt an early progression to a memory T cell phenotype in a cell-intrinsic manner (16–18). Such epigenetic changes have been shown to preserve T cell effector function against acute viral infection and appear to enhance clearance of chronic viral infection (12, 16).

Herein, we examine the effect of *Dnmt3a* gene deletion in donor T cells on alloreactivity using several well-established preclinical murine models of GVHD. We found that BMT with allogeneic T cells lacking *Dnmt3a* resulted in a phenotype of severe systemic and target organ aGVHD consistent across several strain combinations. These findings were associated with early migration and proliferation of T cells in SLOs; potentially reduced apoptosis; and subsequent infiltration into the intestinal tract, a target organ critical to the propagation of the early inflammatory cascade of GVHD (61). Accordingly, serum levels of several proinflammatory cytokines and chemokines associated with GVHD and effector cell migration — including IFN-γ, TNF-α, CXCL10, CCL4, and GM-CSF — were elevated in KO recipients (37, 41, 62–64). By contrast, statistically significant differences in T cell expansion (on days +7 and +14) or cytotoxicity were not observed. As DNA methylation patterns are dependent on the context of cellular activation, a possible explanation for this phenomenon is that these elements may not be restricted by de novo DNA methylation in the inflammatory milieu of a murine model specifically designed to rapidly induce aGVHD.

To our knowledge, this is the first time that the effect of DNMT3a on T cell alloreactivity has been examined in detail. The role of DNA methylation in GVHD and GVT activity has been studied using hypomethylating agents. However, such results are confounded by (i) the fact that pharmacologic DNA methyltransferase inhibition is nonspecific (in terms of both DNMT target enzyme and cell type) and (ii) the well-known cytotoxic effects of these agents (21). By contrast, our work examines the selective effect of DNMT3a — the primary de novo DNA methyltransferase — on donor T cells without the contribution of these confounding factors. Pharmacologic manipulation of activated Th cells with inhibitors such as azacitidine or decitabine alters the normal patterning of gene methylation, resulting in inappropriate coexpression of multiple cytokines and upregulation of FoxP3 (21). Interestingly, azacitidine has demonstrated preclinical activity in attenuating systemic GVHD when administered after experimental BMT, although only in the context of deferring T cell infusion for 11 days after BMT. Moreover, decitabine appeared to be too myelosuppressive in this model (21, 65). The reported effects on GVHD were attributed to relatively enhanced Treg engraftment and generation of inducible Tregs from conventional T cells, with inhibition of proliferation or death of effector T cells (65).

Additionally, we comprehensively characterized the DNA methylome of activated donor T cells after BMT by using genome-wide bisulfite sequencing. In doing so, we employed a powerful analysis tool, informME, which captures both mean methylation levels (MMLs) and methylation variability (stochasticity) (66–68). This tool is highly sensitive and specific in the identification of areas of significant discordance by use of the JSD method of information theory. JSD evaluates distances between probability distributions of methylation, in contrast to conventional analysis based exclusively on mean methylation differences. As previously reported, *Dnmt3a*-KO T cells are able to maintain global distribution of methylation (69). In fact, MMLs appeared to be slightly higher in the KO T cells, potentially due to hypomethylation and overexpression of *Dnmt3b* and *Dnmt1* (data not shown). The distinct alterations that we noted in the epigenome cluster are in areas involved in T cell differentiation and signaling pathways and are relevant to altered gene expression in the experimental group exhibiting severe aGVHD.

An interesting observation in our study was the emergence of a CD4^+^CD8^+^ DP T cell population in the recipients of *Dnmt3a*-KO T cells. Coexpression of CD4 and CD8 is observed in the thymus before thymocytes mature into single-positive T cells that are released into the periphery. After this stage, coexpression of CD4^+^ and CD8^+^ α/β receptors is generally considered to be mutually exclusive and lineage specific, with associated specific MHC restriction for antigen recognition and fixed T cell function (helper vs. cytolytic) (70). Evidence of DP T cells in the periphery has been described in autoimmunity, viral infections (HIV, EBV), and even cancer; however, their prevalence, development, and exact function remain largely unknown and are under investigation (70). To our knowledge, this is the first time that this phenomenon has been described in the context of dysregulated DNA methylation and GVHD in either mice or humans. In our data set, this population has a gene expression profile nearly identical to that of KO CD8^+^ T cells, suggesting that it arises from defective *Cd4* locus silencing. This cell subset did not contribute to increased T cell expansion, and it did not have aberrant or excessive cytokine or cytotoxicity marker production as compared with KO CD8^+^ T cells.

From a translational research perspective, our data draw striking parallels with recent clinical observations: patients with hematological malignancies receiving an allo-BMT from healthy donors with incidental *DNMT3A* mutations (most commonly loss-of-function and associated with asymptomatic CHIP) had higher rates of chronic GVHD, lower risk for relapse, and improved overall survival (14, 15). In these patients, the risk of direct evolution of *DNMT3A*–clonal hematopoiesis to donor cell leukemia (DCL) is low, and most DCLs were traced to atypical donor clonal hematopoiesis involving myelodysplastic syndrome–associated (MDS-associated) genes or germline risk alleles (15). The authors concluded that “only in vivo experiments can decipher mechanisms about how CHIP might lead to GVHD development” (14). Increased aGVHD was not noted in the recipients of grafts harboring *DNMT3A* mutations in this patient cohort, although it was reported in a more recent study (71). In this large, single-institution study, Oran and colleagues examined 363 BMT recipients with acute myeloid leukemia or MDS who received BMT from donors at least 55 years of age, 53 of whom had evidence of CHIP. They found that the incidence of grade II–IV and III–IV aGVHD was significantly higher following BMT with CHIP-positive donors. Importantly, the association remained significant after multivariate analysis. The reason for the discrepancy between these findings and those reported by Frick et al. remains to be determined but may be related to GVHD prophylaxis; 30% of patients in the Frick report received antithymocyte globulin (ATG), whereas none received this therapy in the patient cohort examined by Oran. While severe aGVHD was the prominent finding in our models, we also saw a lesser but notable effect in one model of chronic GVHD (Figure 2F). Obvious differences between the human and murine observations include the fact that all human recipients of allo-BMT receive preemptive GVHD prophylaxis, while the murine models examined herein were designed specifically to induce aGVHD. In addition, although the variant allele frequency of human *DNMT3A* mutations within hematopoietic cells in the clinical studies mentioned above has not yet been described, it is likely that the frequency is not 100%, as in the case in the T cells contained in the graft administered in our experimental models. Similarly, human *DNMT3A* mutations in the context of clonal hematopoiesis are typically heterozygous, in contrast to the complete gene loss in our murine models. Clinical data reporting abrogation of the effect of *DNMT3A* mutations on increased GVHD risk as well as lower risk of malignant relapse after administration of posttransplant cyclophosphamide — which immunomodulates T cell responsiveness (72) — support our hypothesis that this phenomenon is at least partially driven by donor T cells harboring *DNMT3A* mutations.

In the work presented here, DNMT3a appears to provide overarching regulation of T cell alloreactivity in several established murine models of GVHD, via a multitude of pathways. Two examples are presented herein. We found that T cell migration to the intestine was greater in the absence of T cell *Dnmt3a*, a phenomenon mediated at least in part by undermethylation and overexpression of CCR9. Additionally, KO T cells had alterations in epigenetically regulated exhaustion programs, which may have contributed to both enhanced GVHD and augmented antitumor responses. The enhanced GVT activity noted in our experimental model is in accordance with recently published findings of enhanced chimeric antigen receptor (CAR) T cell efficacy after DNA methylation inhibition, either through *Dnmt3a* deletion or pharmacologically (73, 74). Our approach has uncovered multiple other potential mechanisms of allodysregulation, ongoing investigation of which is outside the scope of the current work.

In sum, we demonstrate that de novo DNA methylation is a major mechanism through which alloreactivity is modulated, and our line of investigation has revealed several pathways that could identify potential future therapeutic targets in this context. Our data set may also provide insight into the pathophysiology of a recently described phenomenon in patients receiving allografts from *DNMT3A*-mutated donors; as well as a platform by which these clinical phenomena can be further deciphered to optimize current clinical strategies for mitigating GVHD and optimizing GVT activity following allogeneic BMT.

## Methods

### Mice.

T cell conditional *DNMT3a^2loxp/2loxp^* mice expressing Cre recombinase under control of the *Cd4* promoter were generated on a B6 (H-2^b^) background as previously described (16). Female F1 (H-2^bxd^), BALB/cJ (H-2^d^), B6.C-H2^bm1^/ByJ (Bm1; H-2^b^), and B6(C)-H2^bm12^/KhEgJ (Bm12; H-2^b^) mice aged 8–12 weeks were purchased from the Jackson Laboratory and bred in our animal facility in the Sidney Kimmel Comprehensive Cancer Center of the Johns Hopkins University School of Medicine.

### T cell isolation.

T cells were isolated from splenic single-cell suspensions by positive selection using an autoMACS Pro Separator (Miltenyi Biotec) and anti-CD4, -CD8, -CD90.1, or -CD90.2 magnetic beads as previously described (25, 26, 75). Flow cytometric analysis confirmed the purity of enriched populations, which was greater than 90%. Small intestine T cell isolation was performed as detailed by Sheridan and Lefrançois (76). Lymph nodes were passed through a 70 μm strainer using a 3 cc syringe plunger to generate single-cell suspensions.

### Flow cytometry.

Single-cell suspensions were stained for surface markers and intracellular markers as previously described (24, 25, 75), and data were collected using an Attune NxT Flow Cytometer (Thermo Fisher Scientific) or a BD FACSCelesta Flow Cytometer (BD Biosciences) and analyzed using FlowJo software (Tree Star Inc.).

### BMT experiments.

Recipients were commercially available female mice aged 8–12 weeks. The use of male recipients was avoided, as male aggression, particularly under stress, can negatively (and erroneously) affect experimental end points such as GVHD severity. Donors were female or male, aged 8–12 weeks. WT age- and sex-matched littermates were used as donors serving as controls for all experiments. BMT recipient mice received TBI in 2 fractions using the CIXD Biological Irradiator (Xstrahl Inc.). TBI was given at a total dose of 700–1300 cGy depending on the model, prior to the injection of BM (5 × 10^6^) and purified T cells. The final T cell dose was dependent on the specific murine model employed, as previously described (24, 25, 33, 38, 75) and detailed in Supplemental Table 1. BM and T cells were suspended in 250 μL Leibovitz’s L-15 medium and injected intravenously into the tail vein of recipient mice on day 0 (23–25, 35). Recipient mice were housed in a facility with an acidified water supply; no antibiotics were administered before or after transplant.

### Assessment of aGVHD.

The severity of GVHD was assessed as previously described (24, 25, 75). Recipient mice were ear tagged prior to BMT. Weights were recorded on day 0 and weekly thereafter. Survival was monitored daily. The severity of systemic GVHD was assessed weekly using a semiquantitative scoring system as described previously (77). Tissues were harvested for ex vivo analysis or fixed in 10% buffered formalin for routine histological staining (H&E). Target organ GVHD severity was assessed by histopathology of the tongue, liver, small intestine, and ascending colon in a blinded fashion by a single pathologist, as described previously (23).

### Graft-versus-leukemia experiments.

P815 (H-2K^d^, CD45.2) is a mastocytoma cell line derived from DBA/2 mice (ATCC). Injection of P815 cells into syngeneic mice (H-2K^d^) is uniformly lethal and results in massive tumor infiltration and enlargement of the liver and spleen, with characteristic nodule formation (26). In GVT experiments, B6 WT and *Dnmt3a*-KO mice were used as allo-BMT donors, and 500 P815 tumor cells were added to the BM inoculum on day 0, a time that ensures viable leukemia cells are not affected by the cytotoxic effects of TBI. This GVT mouse model has been instrumental in translational application of novel agents for the treatment or prevention of GVHD (27, 78–83). The P815 cell line was transduced with a lentiviral vector carrying luciferase, allowing visualization of proliferating cells using bioluminescence imaging (BLI). Following intraperitoneal luciferin injection, mice were imaged as described previously (84). BLI images were acquired on an IVIS Spectrum Preclinical In Vivo Imaging System (PerkinElmer) and analyzed with Living Image Software 4.4 (PerkinElmer) as described previously (84). Survival was monitored daily. Tumor burden was assessed weekly by BLI. Postmortem examination as well as spleen and liver weights were also used to confirm the cause of death as either GVHD or tumor (23–25, 75).

### T cell migration experiments.

T cell migration experiments were conducted as previously described (23). Purified B6 WT (CD45.1^+^, CD90.2^+^) and *Dnmt3a*-KO T (CD45.2^+^, CD90.1^+^) cells were stained with CSFE (final 0.5 μM) and eFluor 450 (e450; final 1.25 μM), respectively. The fluorescent dyes used to stain WT and KO cells were reversed in replicate experiments to ensure reproducibility of results. Equal numbers of WT and KO T cells (3 × 10^6^ to 5 × 10^6^) were then coinjected into lethally irradiated F1 mice (CD45.2^+^, CD90.2^+^). Spleens and lymph nodes were isolated from recipient mice at 24, 48, and 96 hours after injection, and lymphocytes were examined by flow cytometry. T cell influx into the small intestine was also evaluated on day +4. Proliferating cells from WT or KO donors were identified based on decreased staining for proliferation dyes, and when cells had lost the fluorescent dye due to ongoing proliferation, they were followed by flow cytometry using the allelic differences in CD45 and CD90 as detailed above.

### MLR assays.

MLR assays were performed as previously described (24, 25). In brief, purified splenic T cells and DCs were suspended in supplemented 10% FBS-RPMI. B6 T cells (2 × 10^5^) were cultured in 96-well plates in the presence of B6 or F1 DCs (2 × 10^4^) at 37°C in a humidified incubator supplemented with 7% CO_2_. Flow cytometry was used to assess per-cell cytokine production and proliferation (24, 25).

### Cytolytic activity.

Cytotoxic T cells (CTLs) were generated as previously described (24, 25, 75). In brief, B6 T cells were stimulated in bulk mixed lymphocyte reaction (MLR) assays in 24-well plates for 4 days. T cells were isolated by Ficoll gradient and added to 96-well plates (starting with 4 × 10^4^ T cells/well and then serial 2-fold dilutions) with radioactive ^3^H-thymidine–labeled P815 (H-2K^d^) or EL4 (H-2K^b^) target cells (1 × 10^3^ cells starting at 20:1 CTL/target ratio). Wells containing target cells only (*T*) were used to determine spontaneous ^3^H release (*S*): *S* = [(*T* – *S*)/*T*] × 100. After 4 hours, cells were harvested from experimental (*E*) wells containing target cells and serially diluted T cells, and assayed for ^3^H-thymidine release. The percentage of cytotoxicity was calculated as [(*S* – *E*)/*S*] × 100.

### WGBS library preparation and sequencing.

WT and KO CD4^+^, CD8^+^, and DP splenic T cells were collected and sorted to greater than 90% purity by flow cytometry on a BD FACSAria on day +10. Genomic DNA was isolated from purified cell populations using a MasterPure DNA Purification kit (New England BioLabs). WGBS single-indexed libraries were generated using a NEBNext Ultra DNA Library Prep kit for Illumina (New England BioLabs) according to the manufacturer’s instructions, with the following modifications: 500 ng input gDNA was quantified by Qubit dsDNA BR assay (Invitrogen) and spiked with 1% unmethylated Lambda DNA (Promega, D1521) to monitor bisulfite conversion efficiency. We fragmented input gDNA with a Covaris S220 Focused-ultrasonicator to an average insert size of 350 bp. Samples were sheared for 60 seconds using Covaris microTUBEs, with instrument settings of duty cycle 10%, intensity 5, and cycles per burst 200. Size selection was performed using AMPure XP beads, and insert sizes of 300–400 bp were isolated. Samples were bisulfite converted after size selection using an EZ DNA Methylation-Gold Kit or EZ DNA Methylation-Lightning Kit (Zymo, D5005, D5030) following the manufacturer’s instructions. After bisulfite conversion, we performed amplification using a KAPA HiFi Uracil+ (KAPA Biosystems, KK282) polymerase based on the following cycling conditions: 98°C 45 seconds/8 cycles: 98°C 15 seconds, 65°C 30 seconds, 72°C 30 seconds/72°C 1 minute. AMPure cleaned-up libraries were run on a 2100 Bioanalyzer (Agilent) High-Sensitivity DNA assay, and samples were also run on the bioanalyzer after shearing and size selection for quality control purposes. We quantified libraries by quantitative PCR (qPCR) using a Library Quantification Kit for Illumina sequencing platforms (KAPA Biosystems, KK4824) and 7900HT Real-Time PCR System (Applied Biosystems). We sequenced WGBS libraries on an Illumina HiSeq4000 instrument using 150 bp paired-end indexed reads and 25% of nonindexed PhiX library control (Illumina). We processed FASTQ files using Trim Galore! version 0.6.4_dev (Babraham Bioinformatics, https://www.bioinformatics.babraham.ac.uk/projects/trim_galore/) to perform single-pass adapter and quality trimming of reads. We aligned reads to the mm10/GRCm38 genome using Bismark version 0.20.0 and Bowtie2 version 2.3.4.2 (http://bowtie-bio.sourceforge.net/index.shtml). We subsequently processed BAM files with Samtools version 1.9 (http://www.htslib.org/) for sorting, merging, duplicate removal, and indexing.

### Differential methylation analysis.

We performed differential analysis between test (KO) and reference (WT) WGBS samples using informME (68). We computed, within analysis regions, JSDs between the corresponding methylation level probability distributions, as well as differences between MMLs (dMMLs) and normalized methylation entropy.

### Genomic features and annotations.

Files and tracks bear genomic coordinates for mm10. We obtained CpG islands (CGIs) from Wu et al. (85). We defined CGI shores as 2 kb sequences flanking CGIs on either side; shelves as 2 kb sequences flanking either side beyond the shores; and open sea as everything else. We used the R package “TxDb.Mmusculus.UCSC.mm10.knownGene” to define genes, exons, and introns. We defined the promoter region of a gene as the 4 kb window centered at its transcription start site (TSS) and determined the gene body region to be the remainder of the gene. We obtained enhancer and promoter annotations, as well as other relevant genomic annotations not mentioned above, using ChromHMM and 15 mm9 mouse chromosomal states defined by Bogu et al. and lifted to the mm10 assembly using the R package “rtracklayer” (86, 87).

### RNA-Seq.

Strand-specific mRNA libraries were generated using the NEBNext Ultra II Directional RNA Library Prep Kit for Illumina, and mRNA was isolated using a Poly(A) mRNA Magnetic Isolation Module (New England BioLabs, E7490). Preparation of libraries followed the manufacturer’s protocol (version 2.2 05/19). Input was 1 μg, and samples were fragmented for 15 minutes for an RNA insert size of approximately 200 bp. The following PCR cycling conditions were used: 98°C 30 seconds/8 cycles: 98°C 10 seconds, 65°C 75 seconds/ 65°C 5 minutes. Stranded mRNA libraries were sequenced on an Illumina HiSeq4000 instrument using 47 bp paired-end dual-indexed reads and 1% of PhiX control. mRNA-Seq depth ranged from 30 to 100 million reads. Reads were aligned to mm10 using STAR version 2.4.2a (88) with the following options: --readFilesCommand zcat --outSAMtype BAM Unsorted SortedByCoordinate --quantMode TranscriptomeSAM GeneCounts. We generated summarized experiment objects using the UCSC mm10 genes.gtf annotation and the following command from the Bioconductor package “GenomicAlignments”: summarizeOverlaps(features=exonsByGene, reads=bamfiles, mode=”Union”, singleEnd=FALSE, ignore.strand=FALSE, fragments=TRUE). Differential expression analysis and significance testing were performed using DESeq2 (89). Following the differential expression analyses, the resulting *t* statistics were run through Wilcoxon’s rank-sum gene set tests using the limma R package on the MsigDB gene sets. Normalized enrichment score (NES) values were generated using the fgsea R package.

### Data availability.

WGBS and RNA-Seq data were deposited in the NCBI’s Gene Expression Omnibus database (GEO GSE196542).

### Statistics.

As previously described (23), survival curves were plotted using Kaplan-Meier estimates. The Mann-Whitney *U* test was used for statistical analysis of in vitro/ex vivo data, clinical scores, and tumor burden, while the Mantel-Cox log-rank test was used to analyze survival data. A *P* value less than 0.05 was considered statistically significant. Analyses were performed using GraphPad Prism version 9.0. Data represent mean ± SEM.

### Study approval.

All animal studies were approved by the Johns Hopkins University IACUC (protocol MO19M300).

## Author contributions

YPK, MAK, CJG, and KRC conceived and designed the study. YPK, MAK, MAB, HHF, AH, ORK, CL, and CJG generated and collected data. YPK, MAK, MAB, HHF, and CJG analyzed data. LL and NJL helped design experiments and interpret data. YPK, MAK, CJG, and KRC contributed to preparation of the manuscript. All authors reviewed and gave final approval of the manuscript.

## Supplementary Material

Supplemental data

## Figures and Tables

**Table 2:**
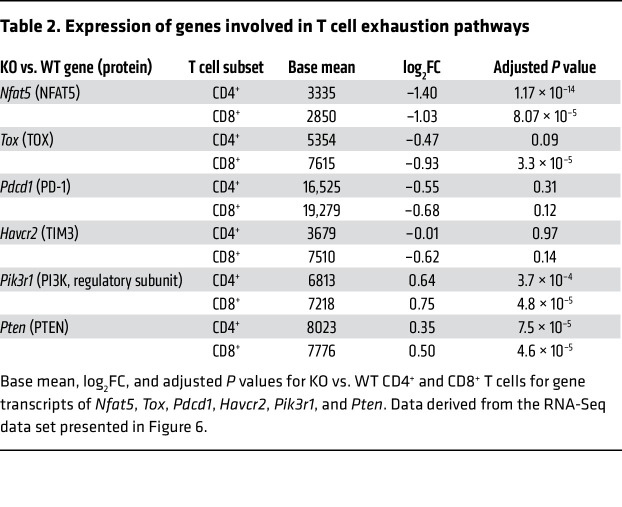
Expression of genes involved in T cell exhaustion pathways

**Table 1:**
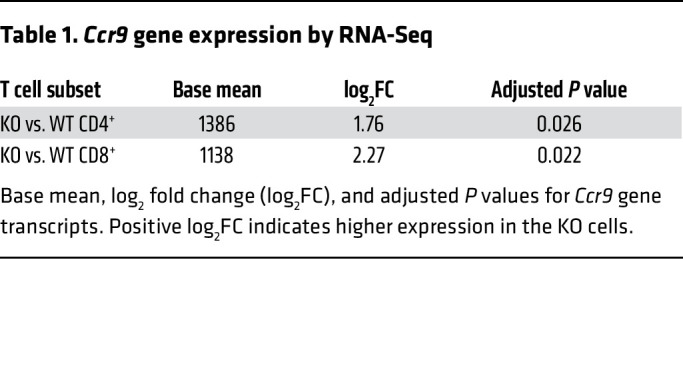
*Ccr9* gene expression by RNA-Seq

## Version 1 05/24/2022

In-Press Preview

## Version 2 07/01/2022

Electronic publication
