# Supplementary material for: Donor T cell DNMT3a regulates alloreactivity in mouse models of hematopoietic stem cell transplantation
Source: J Clin Invest. 2022 Jul 1;132(13):e158047. doi: 10.1172/JCI158047 (PMC9246380; doi:10.1172/JCI158047)
Supplement: Supplemental data [file jci-132-158047-s092.pdf]

Supplemental Figure 1

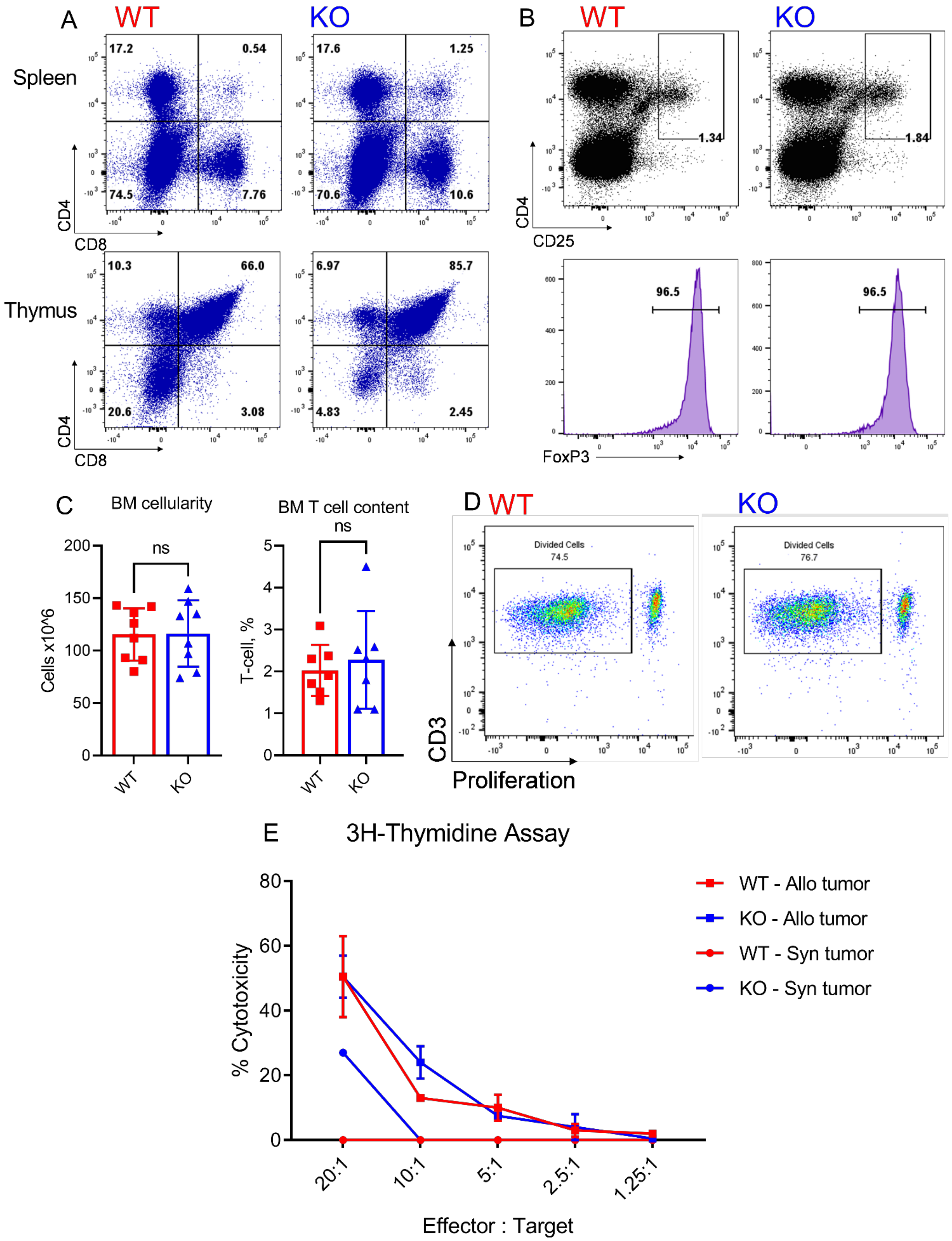

Supplemental Figure 1. WT and DNMT3a T-cell conditional KO donors are immunophenotypically indistinguishable. Representative examples of: A) CD4+ and CD8+ T-cell distribution in the spleen and thymus. B) Treg populations in the spleen as defined by CD4, CD25, intracellular FoxP3 expression. C) Bone marrow cellularity (n=8 per group) and bone marrow T-cell content (n=5 per group). D) Proliferation as a response to allogeneic dendritic cell stimulation in a mixed lymphocyte reaction (MLR) as described in Methods. E) Cytotoxicity in a JAM assay by thymidine incorporation following activation bulk MLR as described in Methods.

Supplemental Figure 2

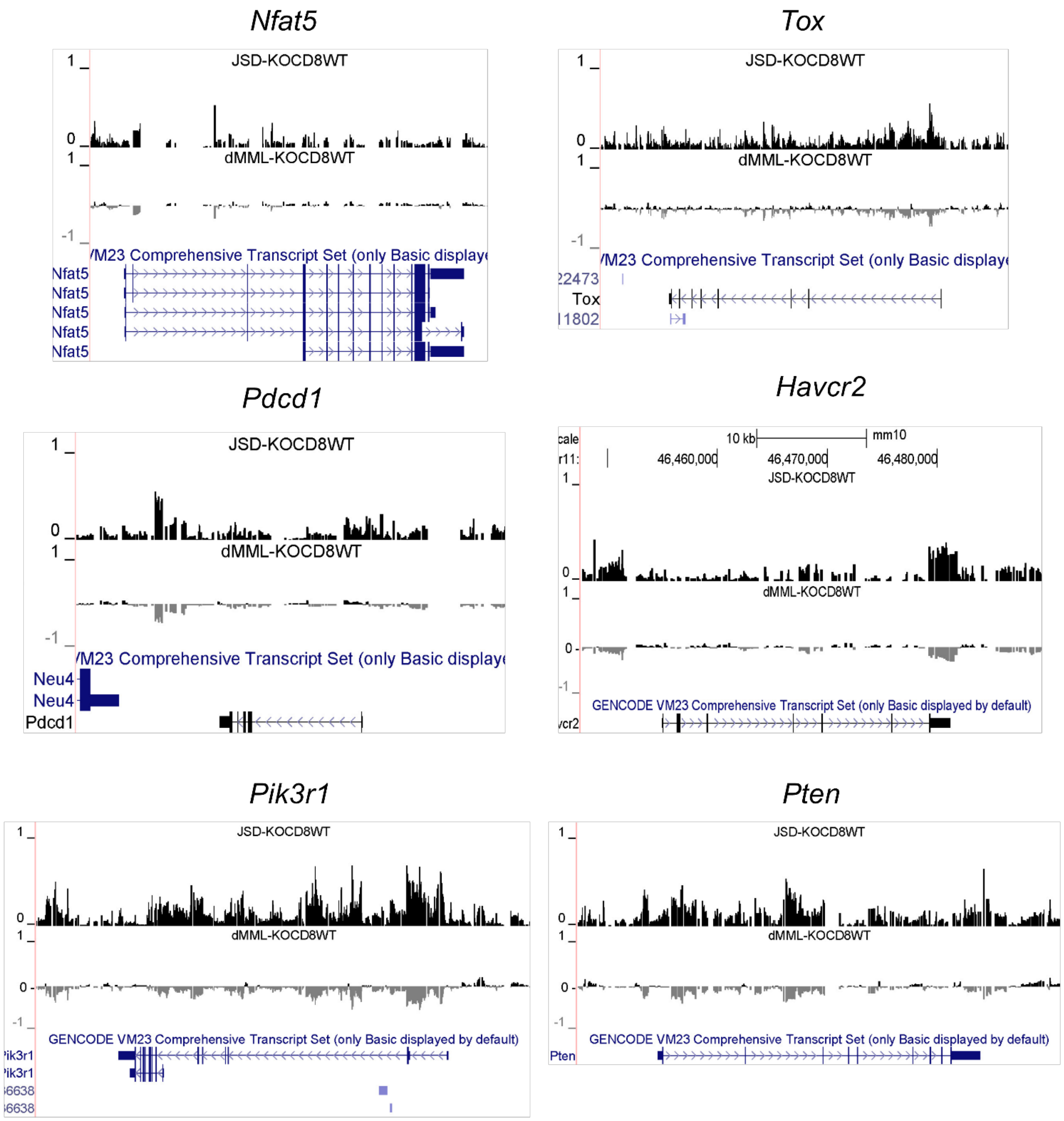

Supplemental Figure 2. JSD and dMML of genes presented in Figure 7C.

Supplemental Figure 3

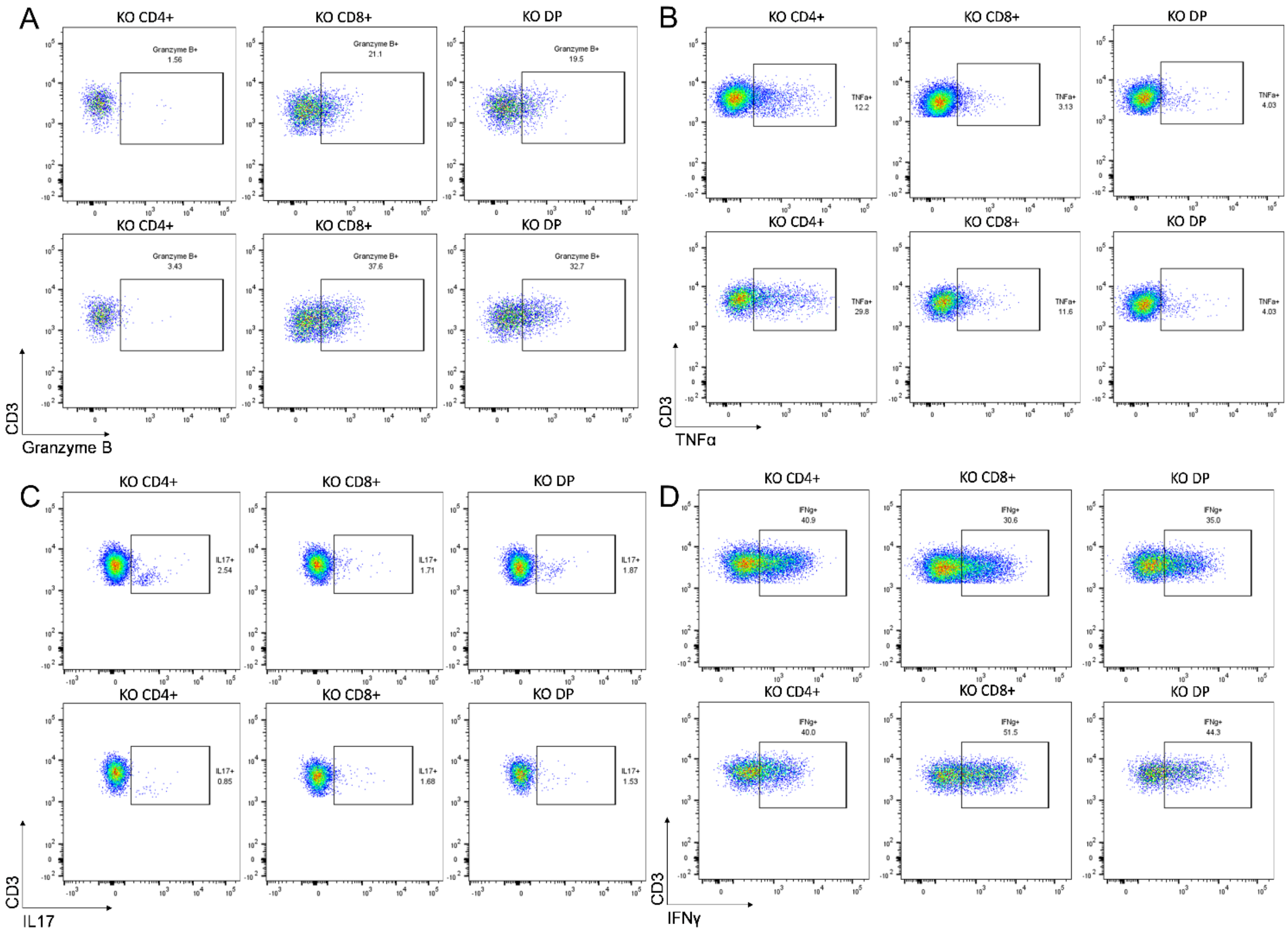

Supplemental Figure 3. CD4+CD8+ double positive KO T-cells have similar expression profiles to KO CD8+ T-cells. Representative examples of flow-cytometry based expression of splenic KO CD4+, KO CD8+, and KO CD4+CD8+ T-cells at day +7 post-BMT of the following markers: A) Granzyme B, B) TNFa, C) IL17, D) IFNγ.

Supplemental Figure 4

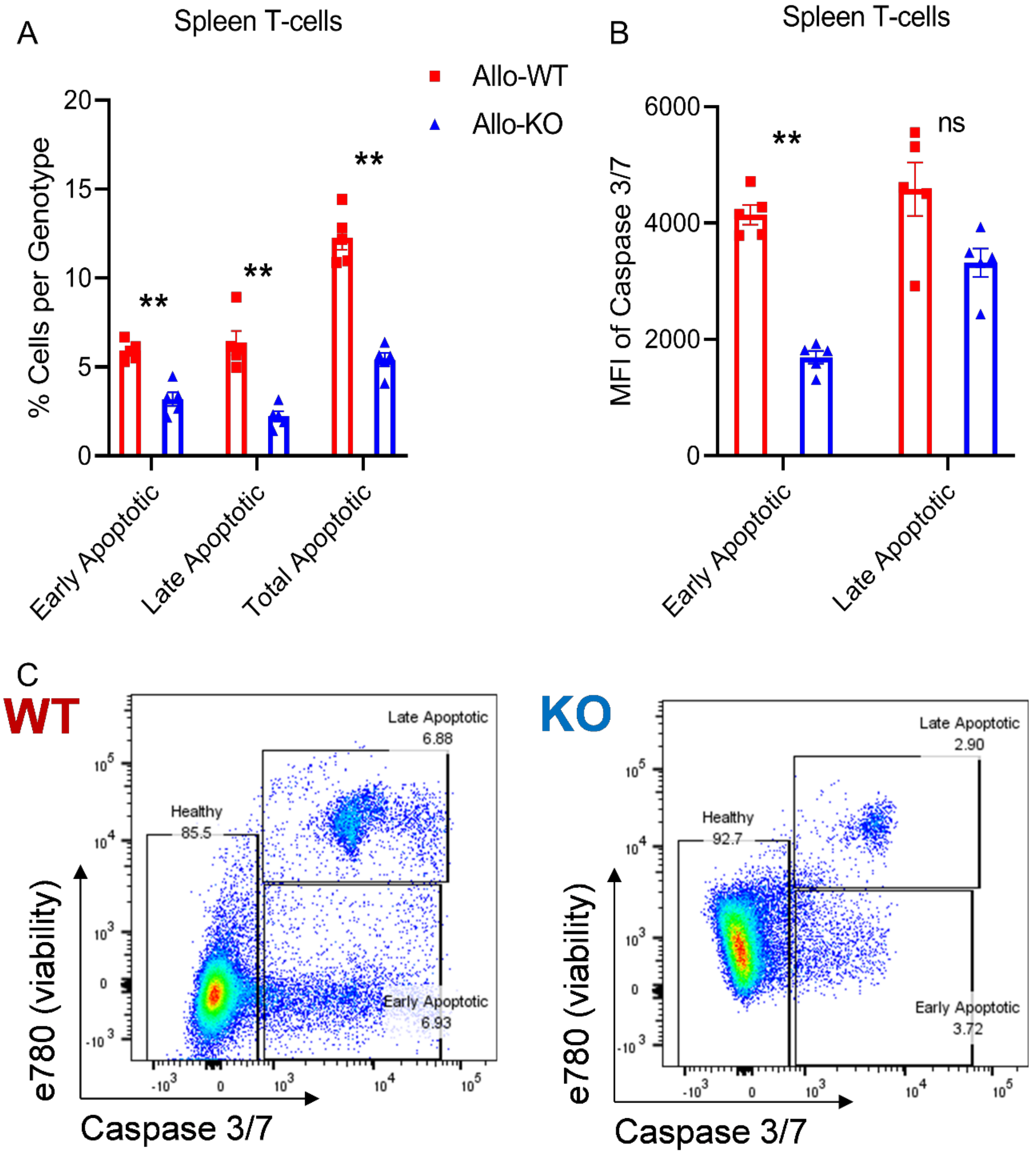

Supplemental Figure 4. Lack of DNMT3a in donor T-cells is associated with lower expression of caspase 3/7. Flow-cytometric caspase-3/7 assay as a marker of apoptotic activity in splenic T-cells, 48-hours after co-adoptive transfer (using the model presented in Figure 4). Results shown are representative of two replicate experiments, n=5 each. A) Percentages of early, late, and total apoptotic cells within the WT and KO splenic T-cell populations B) Median fluorescent intensity (MFI) of caspase 3/7 C) Examples of flow-cytometry plots. *$p<0.05$ with Mann-Whitney *U* test.

Supplemental Table 1. Experimental parameters of murine models of allo-BMT.

**Donor**	**Recipient**	**Mismatch**	**TBI (cGy)**	**BM cells**	**T-cells**
C57BL/6J<br>(B6; H-2^b)	B6D2F1<br>(F1; H-2^{bxd})	Major (haploidentical)	1300	5 x 10^6	1-2 x 10^6
C57BL/6J<br>(B6; H-2^b)	BALB/cJ<br>(Balb, H-2^d)	Major	700	5 x 10^6	0.5-1 x 10^6
C57BL/6J<br>(B6; H-2^b)	B6.C-H2<bm1>/ByJ<br>(Bm1; H-2^b)	Major	1300	5 x 10^6	2 x 10^6 CD8+
C57BL/6J<br>(B6; H-2^b)	B6.C-H2<bm12>/KhEgJ<br>(Bm12; H-2^b)	Major	900-1100	5 x 10^6	2.5 x 10^5 CD4+

Supplemental Table 1. Experimental parameters of murine models of allo-BMT.

Supplemental Table 2. JSD of KO vs WT CD4+ T-cells, ranked in descending order.

Rank	Gene	Rank	Gene	Rank	Gene	Rank	Gene
1	Anks1	76	Ramp1	151	Abl1	226	Art4
2	Rnf157	77	Trp53i13	152	Bcat1	227	P2ry14
3	Txnip	78	Enthd1	153	Mlec	228	Rexo1
4	Ncor2	79	Irf8	154	Ikzf2	229	Adora2a
5	Uba7	80	Mad1l1	155	Phldb3	230	Tle6
6	Rab3ip	81	Abcb9	156	Rapgef3	231	Sik1
7	Cbfa2t3	82	Abhd15	157	Psma6	232	Setd4
8	Tcf7	83	Rnf43	158	Mmp9	233	Mtch1
9	Gpr146	84	Pbx2	159	Rasl11b	234	Myl12b
10	Cmtm7	85	Nyap1	160	Zfp236	235	Atg16l2
11	Vps37b	86	Jakmip1	161	Dhrs3	236	Ptpn6
12	Lyl1	87	Pitpnm2	162	Ptms	237	Arhgap45
13	Gm15441	88	Aqp11	163	Fam189b	238	Mgat4a
14	Foxp1	89	Hsdl1	164	Vrk1	239	Gm9530
15	Dnmt3a	90	Gpr132	165	Ccr9	240	Frmd4a
16	Patz1	91	Ly6m	166	Lag3	241	Slc38a6
17	Znrf1	92	Stat3	167	Nr3c1	242	Zyx
18	Ptp4a3	93	Tmem63a	168	Zmynd8	243	Slc29a1
19	Stat5b	94	Gngt2	169	Muc13	244	Otx1
20	Cux1	95	Mir7687	170	Il1r1	245	Fam71b
21	Agtrap	96	Ppp1r21	171	Erf	246	Sema4a
22	Hdac7	97	Pxn	172	Emp3	247	Mical3
23	Dtx1	98	Plekho1	173	Dnajc7	248	Bcl9
24	Nr4a3	99	Tbxa2r	174	Sestd1	249	Lcp2
25	Slc16a5	100	Smad7	175	Ftl1	250	Crtc3
26	Satb1	101	Endou	176	Arpc3	251	Nfam1
27	Chdh	102	Fam78a	177	Tlr6	252	Rai1
28	Il17rb	103	Rabgap1l	178	Vax2os	253	Fmnl3
29	Inka1	104	Slc43a2	179	Lrp5	254	Trim13
30	St6gal1	105	Hps4	180	Rgs3	255	Ripor2
31	Smim5	106	Eya2	181	Edem1	256	Eomes
32	Epg5	107	Tom1l2	182	9230102O04Rik	257	Zfp219
33	5830418P13Rik	108	Mvb12b	183	Rara	258	Uck2
34	Emilin1	109	Ldhd	184	Pacsin2	259	Pdk1
35	Ccm2	110	Runx3	185	Chd7	260	Ankrd13a
36	Auh	111	Hpcal1	186	Rapgef2	261	Pdlim4
37	Cyren	112	Fkbp5	187	Rmnd5a	262	Csnk1e
38	Fam102a	113	Tcf25	188	Sh3rf1	263	Lipe
39	Rps6ka1	114	Fgr	189	Flt3l	264	Ets2
40	5830428M24Rik	115	Gm11346	190	Slc37a3	265	Ccdc162

41	Egr3	116	St3gal2	191	Ppp1r9b	266	Wasf2
42	Dntt	117	Neurl3	192	Commd3	267	Nsmce1
43	Dot1l	118	Gne	193	Plekhh3	268	Gramd3
44	Pgpep1l	119	Med24	194	Csad	269	Rbms2
45	Kif23	120	Zfp652	195	Ggta1	270	Lgals7
46	Rnf166	121	Trak1	196	Ezr	271	Tnik
47	Il6st	122	Heg1	197	Cd72	272	Relt
48	Smad3	123	Tapt1	198	Fmnl1	273	Gemin5
49	Sidt2	124	Ccdc12	199	Map7d1	274	Auts2
50	Zfp710	125	Aldh3b1	200	Mir23a	275	Amz2
51	Klhl3	126	Poc1b	201	Pik3cd	276	Ssbp2
52	Psap	127	Nomo1	202	Pde4d	277	Jade1
53	A430093F15Rik	128	Sytl3	203	Mir24-2	278	Dnajc6
54	5033406O09Rik	129	Polm	204	Fut7	279	Vgll4
55	Ppa2	130	Ubxn11	205	Mir3074-2	280	Plxnc1
56	Mdk	131	Cdk5rap1	206	Gm12216	281	Git2
57	Notch1	132	Stk39	207	Elovl6	282	Tle3
58	Xrcc6	133	Stam	208	Sapcd1	283	Rrp1
59	Foxn3	134	Ssbp3	209	Scmh1	284	Fos
60	Smim6	135	Retreg1	210	Gata3	285	Bach2
61	Kdm6b	136	Zfp740	211	Parp11	286	Nfatc2
62	Fes	137	Aopep	212	Ttc13	287	Csrnp1
63	Plac8	138	Rassf2	213	Chrna9	288	Id3
64	Ankrd28	139	Adgrg1	214	Gng10	289	Socs5
65	Hic1	140	C730014E05Rik	215	Mir27a	290	4732471J01Rik
66	Gm15880	141	Trmt1	216	Nim1k	291	Angpt2
67	Tle5	142	Susd1	217	Arpp21	292	Stk4
68	Stat1	143	Lgals9	218	BC049352	293	Gm19705
69	Lrba	144	Sema4b	219	Capn3	294	Tcf4
70	Lck	145	4930412O13Rik	220	Chd3	295	Cers6
71	Mab21l2	146	Vezf1	221	Bex6	296	Acot7
72	Eef2k	147	Med13l	222	Gm4632	297	Sema4d
73	2610307P16Rik	148	Lrig1	223	Ikzf3	298	Sgsh
74	Rgs10	149	Rreb1	224	Peg12	299	Plcg1
75	Gtf2i	150	Sgk3	225	Col16a1	300	H60b

Supplemental Table 2. Genes (top 300) identified to be differentially methylated between KO and WT CD4+ T-cells. Higher JSD values (0-1) indicate differences in methylation due to differences in mean methylation level, methylation entropy, or both.

Supplemental Table 3. JSD of KO vs WT CD8+ T-cells, ranked in descending order.

Rank	Gene	Rank	Gene	Rank	Gene	Rank	Gene
1	Anks1	76	Vezf1	151	Mir23a	226	Aqp11
2	Ncor2	77	Dntt	152	Sell	227	Vrk1
3	Tcf7	78	5830428M24Rik	153	Mir1190	228	Cd47
4	Rnf157	79	Gm15880	154	Mir24-2	229	Zfp652
5	Uba7	80	Ramp1	155	Mir3074-2	230	Gata3
6	Ccm2	81	Gngt2	156	Fam167b	231	Relt
7	Txnip	82	Lck	157	Tcirg1	232	Dzip1
8	Cbfa2t3	83	Slc43a2	158	Lcp2	233	Nfe2l2
9	Patz1	84	Tmie	159	Ankrd13a	234	Insl3
10	Vps37b	85	Scn2b	160	Cd3e	235	Wnt10b
11	Fam78a	86	Epg5	161	Lasp1	236	Ezr
12	Stat3	87	Susd1	162	Ubxn11	237	Nfatc2
13	Kif23	88	Als2cl	163	Tspan13	238	Fmnl1
14	Rab3ip	89	A430093F15Rik	164	Elmo3	239	Mir7023
15	Satb1	90	Med24	165	Med13l	240	Tlr12
16	Foxp1	91	Sidt2	166	Zbtb34	241	Rnf43
17	Adgrg1	92	Sh3rf1	167	Abhd15	242	Klf3
18	Gpr146	93	Rab11fip4os2	168	5033406O09Rik	243	Zbtb7b
19	Stat1	94	Ldlrap1	169	Zmynd8	244	Cnr2
20	Slc16a5	95	Mad1l1	170	Aopep	245	Poc1b
21	Adgrg5	96	Tapt1	171	Fmnl3	246	Frmd4b
22	Dnmt3a	97	Nsmce1	172	Eef2k	247	Sun2
23	Arhgap45	98	Vax2os	173	Plac8	248	Mpzl2
24	Smim5	99	Tcf25	174	Ptms	249	Scx
25	Neurl3	100	Fam189b	175	Slc2a3	250	Map7d1
26	Psap	101	Tle3	176	Pik3cd	251	Slfn8
27	Znrf1	102	Wasf2	177	Sgsh	252	Hpcal1
28	Xrcc6	103	Emilin1	178	Trp53i13	253	Ttc7
29	Egr3	104	Rassf2	179	Grb7	254	Ccdc162
30	Fes	105	Ccr9	180	Lgals9	255	Dnajc7
31	Notch1	106	Tle5	181	Rreb1	256	Aldh3b1
32	Gm15441	107	Pvt1	182	Myh9	257	Il1r1
33	Pxn	108	Hic1	183	Plcg1	258	Lipe
34	Dot1l	109	Smad7	184	Lfng	259	Jaml
35	Rnf166	110	Tnfrsf1a	185	Pde2a	260	Rbms2
36	Ly6m	111	St6gal1	186	2610307P16Rik	261	Polg
37	Hsdl1	112	Smad3	187	Ldhd	262	Gpr25
38	Cux1	113	Kif21b	188	Trim13	263	Mab21l2
39	Jakmip1	114	Bcat1	189	Sytl3	264	Pkn1
40	Ptpn6	115	Ptp4a3	190	Galnt6	265	Rasgrp1

41	Ppa2	116	Rrp1	191	Gramd3	266	Rara
42	Gtf2i	117	Zfp740	192	Pitpnm2	267	Gm35584
43	Endou	118	Eya2	193	Polm	268	Tle4
44	Rps6ka1	119	Rarg	194	Bach2	269	Ripor2
45	Nr4a3	120	Tspan2os	195	Art4	270	Pacsin2
46	Cmtm7	121	Gpr132	196	Acp5	271	Sh3bp5
47	5830418P13Rik	122	Tom1l2	197	Kcna2	272	Prex1
48	Adcy7	123	Chd7	198	Flt3l	273	Fgr
49	Cyren	124	Rabgap1l	199	Rexo1	274	Slc37a3
50	Il6st	125	Mir7674	200	Itpr1	275	Lbh
51	Abl1	126	Nup214	201	Ndst1	276	Rcbtb2
52	Gm11346	127	Abcb9	202	Bop1	277	Gm9530
53	Nfkb1	128	Bex6	203	Plekhh3	278	Aff1
54	Mir7687	129	Pgpep1l	204	Nyap1	279	Arpc3
55	Rgs3	130	Ccdc12	205	Edem1	280	Uck2
56	Inka1	131	Chdh	206	Muc13	281	Rnf213
57	Trak1	132	Heg1	207	Mir8119	282	Scml4
58	Ggta1	133	Pdk1	208	Scd2	283	BC051537
59	Stat5b	134	Plekho1	209	Fkbp5	284	Psma6
60	Lyl1	135	Nomo1	210	Wnt10a	285	Zeb1
61	Stk39	136	Fam71b	211	Vgll4	286	Chd2
62	Ssbp3	137	Il17rb	212	G0s2	287	Ifitm1
63	Mlec	138	Fam53b	213	Hdac7	288	Cdk5rap1
64	Rmnd5a	139	Cd2	214	Stam	289	Spata13
65	Irf8	140	Gng10	215	Nim1k	290	Paqr7
66	Enthd1	141	Agtrap	216	Tlr6	291	Capn3
67	Nr4a2	142	Tspan2	217	Tmc8	292	Fut7
68	Foxn3	143	Gm15850	218	Gm12216	293	4732471J01Rik
69	Fam102a	144	Nr3c1	219	Niban2	294	Upb1
70	Fchsd2	145	Gne	220	Abi3	295	Rere
71	Kctd10	146	Lncppara	221	Dtx1	296	Col16a1
72	Bcl9l	147	Parp11	222	Jade1	297	Prrt1
73	Rgs10	148	Mir27a	223	Pbx2	298	Cd4
74	Mdk	149	Crtc3	224	Smim6	299	Kdm6b
75	Auh	150	Stk4	225	Unc13d	300	Ttc39b

Supplemental Table 3. Genes (top 300) identified to be differentially methylated between KO and WT CD8+ T-cells. Higher JSD values (0-1) indicate differences in methylation due to differences in mean methylation level, methylation entropy, or both.

Supplemental Table 4. Differentially expressed genes in KO vs WT CD4+ T-cells.

Gene	Base mean	Log2FC	*p*-adj	Gene	Base mean	Log2FC	*p*-adj
Trnp1	54.0	4.37	0.012	Hspa1b	1885.4	-7.26	0.000
Cd79b	434.8	3.72	0.000	Hspa1a	3152.8	-7.24	0.000
Gzma	7733.1	3.49	0.000	Actbl2	64.7	-4.77	0.038
Emilin1	235.6	3.17	0.000	Fosb	957.3	-4.38	0.000
G0s2	81.4	2.95	0.000	Pklr	68.8	-3.56	0.000
Gm15708	112.4	2.78	0.000	Krt17	136.2	-3.43	0.028
Cabp1	65.0	2.68	0.000	Apol11b	928.5	-3.31	0.016
Scn2b	127.1	2.67	0.000	Unc13b	190.8	-3.26	0.006
Pygm	312.7	2.64	0.000	Strc	50.0	-3.20	0.000
Qrfp	853.6	2.57	0.000	Sspo	64.6	-3.20	0.000
Lrp5	875.7	2.53	0.000	Snca	122.8	-3.04	0.024
Igsf23	296.8	2.49	0.000	Gcg	1763.4	-2.99	0.000
Ston1	371.6	2.42	0.000	Fos	2343.4	-2.95	0.000
Sorcs2	56.1	2.25	0.001	Ttn	791.9	-2.82	0.000
Gpnmb	960.7	2.25	0.000	Scin	261.4	-2.75	0.000
5830418P13Rik	58.2	2.24	0.000	Gm16712	90.4	-2.72	0.000
Klrb1c	405.8	2.23	0.003	Jun	2570.9	-2.70	0.000
Muc13	54.9	2.15	0.022	Dmxl2	72.6	-2.68	0.000
Fes	1557.1	2.14	0.000	Otx1	56.8	-2.67	0.001
Gpr25	141.5	2.13	0.030	Miat	459.0	-2.62	0.000
Haao	307.2	2.10	0.000	Hpn	71.1	-2.59	0.000
Cnr2	175.1	2.07	0.000	Naip5	98.8	-2.58	0.000
Rcn3	181.8	2.06	0.000	Gatm	177.7	-2.55	0.000
Aqp9	265.5	2.02	0.000	Tmcc2	241.4	-2.55	0.046
Txnip	28545.0	1.98	0.006	Ciita	129.4	-2.53	0.000
Atp1b1	98.3	1.96	0.001	Plbd1	222.6	-2.51	0.000
Platr17	90.6	1.94	0.000	Il20ra	53.6	-2.50	0.003
Alpk2	74.9	1.92	0.007	Rab7b	58.8	-2.49	0.000
Apobec2	807.0	1.88	0.000	Aldh1a1	101.1	-2.43	0.001
Fam109b	141.0	1.85	0.000	Cxcl9	150.8	-2.38	0.000
Gp2	78.3	1.83	0.011	Synpo2	56.9	-2.35	0.000
Fcer1g	339.9	1.80	0.000	Zbtb46	118.6	-2.26	0.000
Pgam2	114.3	1.80	0.000	Prrt2	74.8	-2.24	0.000
Ccr9	1385.9	1.76	0.026	Cd83	403.4	-2.23	0.000
Ankrd35	93.3	1.73	0.002	Malat1	24158.8	-2.23	0.000
Palm	941.5	1.72	0.000	Lpl	118.5	-2.20	0.000
Tmem176b	1233.9	1.72	0.001	Slamf8	52.3	-2.18	0.000
Adgrg5	168.4	1.70	0.000	Mmp9	657.7	-2.17	0.000
Pls1	54.5	1.70	0.001	Aif1	122.8	-2.17	0.000
Wfikkn2	2485.1	1.68	0.000	Sema6d	64.8	-2.17	0.000

Nrarp	507.8	1.67	0.000	Hk3	270.1	-2.16	0.000
Rras2	1834.7	1.66	0.000	Ppfibp2	72.3	-2.16	0.002
Gm4285	159.7	1.65	0.000	Mir155hg	86.6	-2.15	0.000
Aipl1	71.0	1.63	0.021	Anpep	56.8	-2.14	0.018
Pik3ip1	2735.1	1.61	0.001	Ppp1r15a	3163.9	-2.13	0.000
Tusc1	91.2	1.60	0.001	Insm1	86.4	-2.13	0.003
Ifitm3	4234.7	1.59	0.000	Snora81	100.6	-2.12	0.000
Amica1	871.7	1.58	0.000	Clgn	184.2	-2.12	0.000
C1qtnf4	60.0	1.58	0.030	Wdfy4	441.7	-2.12	0.006
D930028M14Rik	92.9	1.57	0.002	Samd3	143.7	-2.09	0.000
Tmem176a	828.3	1.51	0.010	Nlrp1b	69.8	-2.06	0.000
Fam212a	150.8	1.50	0.000	Myof	64.4	-2.06	0.001
9030617O03Rik	503.9	1.49	0.000	Ltk	71.0	-2.06	0.001
Grb7	826.1	1.48	0.000	Apol10b	121.3	-2.05	0.000
Cerk	708.5	1.47	0.000	Clec7a	62.4	-2.03	0.017
2810001G20Rik	679.2	1.47	0.000	Itga2b	52.0	-2.02	0.000
Cpm	488.9	1.46	0.000	Samd14	71.9	-2.00	0.010
Zfp112	51.6	1.46	0.015	Col18a1	52.4	-1.98	0.000
Lrrc25	91.1	1.44	0.000	Ccdc184	94.1	-1.96	0.000
Wbscr27	79.2	1.44	0.005	Myo1h	78.0	-1.96	0.006
Irak3	1153.1	1.43	0.000	H2-Eb1	904.2	-1.95	0.000
Gm16845	135.8	1.42	0.001	6330409D20Rik	52.0	-1.94	0.003
Gzmb	33210.3	1.41	0.003	Prpf40b	50.5	-1.94	0.002
Try4	95.8	1.41	0.027	Trp63	79.4	-1.93	0.006
Hif3a	142.7	1.40	0.049	Degs2	345.8	-1.93	0.000
Sh3pxd2a	198.6	1.40	0.000	Plk2	369.7	-1.93	0.005
1810041H14Rik	106.8	1.39	0.000	Mir142	60.6	-1.89	0.001
Gm19705	180.8	1.38	0.000	Pisd-ps1	975.6	-1.88	0.000
Snhg4	445.0	1.36	0.000	Ptpn5	157.1	-1.87	0.000
A630066F11Rik	90.8	1.36	0.017	Tns3	120.7	-1.87	0.000
Npc1	3516.3	1.36	0.000	Abcb4	71.8	-1.85	0.024
Dnajc6	500.1	1.35	0.000	St3gal5	205.6	-1.85	0.000
Cd276	148.2	1.33	0.039	Insl3	97.5	-1.84	0.000
Cpa1	151.3	1.33	0.038	Atp1b2	50.7	-1.84	0.020
Gm17745	520.9	1.32	0.000	H2-Aa	829.0	-1.84	0.009
Gng3	103.1	1.32	0.000	Mpo	86.9	-1.83	0.041
Susd1	95.6	1.31	0.000	Rasd2	82.4	-1.83	0.000
Ifitm1	3775.5	1.30	0.047	Mir7115	86.0	-1.81	0.000
Ppp1r3b	135.7	1.30	0.000	Ces2d-ps	199.8	-1.80	0.001
Zfp580	139.0	1.30	0.007	Syna	65.6	-1.80	0.003
Slc41a2	87.6	1.30	0.003	Lmo2	101.8	-1.79	0.013

Il7r	12144.2	1.29	0.000	Gm10825	80.0	-1.79	0.010
Gpr146	1595.3	1.28	0.000	F13a1	63.6	-1.78	0.025
Bbc3	1951.7	1.27	0.017	Slc30a3	91.1	-1.77	0.004
Dtx1	9434.6	1.27	0.024	Gda	271.0	-1.76	0.020
Apold1	53.8	1.26	0.040	Sulf2	71.8	-1.76	0.001
Il20rb	197.5	1.25	0.003	Lima1	67.3	-1.76	0.001
Cdnf	90.3	1.25	0.015	Tbx6	64.7	-1.74	0.006
Fkbp5	23039.1	1.24	0.000	Hapln1	104.5	-1.74	0.001
Serpinf1	250.9	1.24	0.009	Vwa5a	567.9	-1.73	0.000
Cirbp	3717.2	1.24	0.017	Trpm2	62.0	-1.73	0.004
Abcb9	2682.0	1.24	0.000	Hck	108.8	-1.73	0.000
Smo	63.2	1.23	0.014	Snord22	91.1	-1.73	0.000
Fam120aos	489.6	1.23	0.000	Alox5ap	56.9	-1.71	0.018
Ttyh3	5877.9	1.22	0.004	Oplah	57.6	-1.70	0.008
Trib3	150.5	1.22	0.004	Hebp1	105.0	-1.70	0.000
Rnls	75.3	1.20	0.023	Rab3il1	91.7	-1.69	0.000
2310039H08Rik	341.2	1.20	0.000	Olfml2a	110.8	-1.69	0.006
Tmem9	1076.8	1.20	0.000	Tspan33	82.6	-1.69	0.044
1700096K18Rik	116.0	1.19	0.010	Spi1	144.4	-1.68	0.000
F2rl1	1014.2	1.19	0.000	Hist1h1d	117.8	-1.68	0.002
Rom1	2297.0	1.19	0.015	Palld	57.2	-1.67	0.003
Cacnb4	50.5	1.19	0.045	Slc2a4rg-ps	1008.4	-1.67	0.000
D330050I16Rik	58.0	1.19	0.044	Cd74	3456.2	-1.67	0.000
Hid1	2477.8	1.15	0.000	Gucy2e	55.3	-1.66	0.022
Lgals3	10475.1	1.15	0.000	Rassf4	105.5	-1.64	0.000
Prdm16	181.0	1.15	0.001	Mpeg1	811.9	-1.62	0.000
Mt1	555.4	1.15	0.006	Dnmt3a	3259.3	-1.62	0.000
Hsf4	269.4	1.14	0.000	Gabbr1	973.5	-1.62	0.000
Mgst2	190.0	1.14	0.023	Dusp1	3061.2	-1.61	0.002
Cand2	832.0	1.14	0.000	Tppp3	60.7	-1.60	0.002
Tgfb3	625.3	1.13	0.001	Pisd-ps2	299.6	-1.60	0.000
Ndrg1	1202.5	1.13	0.000	Tvp23a	78.2	-1.60	0.000
Nmnat2	148.4	1.13	0.005	Hemgn	145.4	-1.59	0.019
Ifitm2	4118.6	1.13	0.000	Cd70	366.3	-1.59	0.000
Zbtb11os1	137.1	1.12	0.001	Prr33	349.6	-1.59	0.000
Tbxa2r	991.6	1.11	0.000	Nav2	622.6	-1.58	0.000
Gp1ba	71.8	1.11	0.042	Clec12a	64.8	-1.58	0.001
Dirc2	502.2	1.11	0.001	Rasgef1a	143.3	-1.58	0.001
Bcl2	10412.1	1.11	0.000	Pirb	67.2	-1.58	0.001
Dnmt3b	1113.9	1.10	0.000	Tnc	54.1	-1.58	0.009
Sestd1	458.0	1.10	0.000	Kit	124.8	-1.57	0.000

Cpt1a	8486.1	1.10	0.013	Ptprv	1075.1	-1.57	0.002
N4bp2	1550.5	1.08	0.000	Card10	249.5	-1.56	0.000
Magi2	58.2	1.08	0.020	Blvrb	284.5	-1.56	0.000
Slc14a1	793.8	1.08	0.000	Rhd	62.7	-1.55	0.031
Pde2a	3692.5	1.07	0.000	Ly86	54.5	-1.55	0.002
1500009L16Rik	1313.3	1.07	0.001	Myo1d	74.7	-1.55	0.027
Tmem203	593.5	1.07	0.000	Ptgs1	78.1	-1.54	0.001
Plac8	8895.4	1.07	0.000	4930579G18Rik	175.6	-1.54	0.000
Rab3d	1606.6	1.07	0.000	Csf2ra	100.7	-1.53	0.000
Spef2	87.4	1.07	0.021	Lppr3	276.0	-1.53	0.000
Ldhb	861.9	1.06	0.002	Arnt2	726.1	-1.52	0.000
Lat2	287.4	1.05	0.000	Cacng8	82.8	-1.52	0.005
Ldlrap1	1982.9	1.05	0.000	Trim2	73.1	-1.52	0.001
Extl2	343.3	1.05	0.000	Trp73	395.3	-1.51	0.000
Pdcd4	12562.8	1.04	0.000	Cav2	99.5	-1.50	0.002
D130017N08Rik	60.9	1.04	0.027	Plcd1	85.7	-1.50	0.001
Tomt	107.2	1.04	0.011	Tbc1d9	80.2	-1.50	0.007
Gm4890	55.5	1.04	0.025	Cadm1	106.8	-1.50	0.007
1700056E22Rik	110.6	1.04	0.007	Snora64	82.4	-1.50	0.001
Tbc1d16	124.0	1.03	0.001	C4b	61.7	-1.49	0.001
Eps8	319.0	1.03	0.002	Vipr2	76.1	-1.48	0.007
Tef	1081.2	1.03	0.001	App	231.9	-1.48	0.000
Dyx1c1	237.3	1.03	0.008	Ablim2	72.9	-1.48	0.003
Galnt4	1413.0	1.02	0.000	Gm20605	292.9	-1.47	0.000
Camk2b	2540.0	1.02	0.000	Susd2	1677.8	-1.47	0.000
Vmac	455.1	1.02	0.002	Ubd	108.5	-1.47	0.001
Cd72	186.2	1.01	0.042	A930013F10Rik	101.8	-1.47	0.002
Klhl5	1411.2	1.01	0.000	B4galt4	79.9	-1.47	0.001
Sft2d3	309.9	1.01	0.002	Ctsh	78.7	-1.46	0.001
Fbxo17	182.7	1.00	0.031	Igsf9b	103.5	-1.46	0.003
H6pd	1775.8	1.00	0.000	H2-Ab1	1080.2	-1.46	0.003
Wdr34	145.1	1.00	0.002	Nid2	125.2	-1.46	0.000
Acp5	6388.2	0.99	0.000	Slc43a1	301.2	-1.45	0.002
Ddit4	18173.3	0.99	0.000	Nr1h3	117.5	-1.45	0.025
Magef1	236.0	0.99	0.010	Shtn1	202.1	-1.45	0.000
Macrod1	190.8	0.99	0.000	Tifab	69.6	-1.44	0.021
Zbed3	473.4	0.98	0.001	Mir3064	148.6	-1.44	0.002
Snhg3	356.4	0.98	0.007	Igfbp7	1292.1	-1.43	0.016
Mblac1	78.7	0.98	0.044	Atat1	72.2	-1.43	0.002
C1qtnf6	792.0	0.97	0.000	Tnfaip2	206.7	-1.43	0.005
Klf2	11419.5	0.97	0.000	Phlda1	788.9	-1.43	0.000

Sprn	107.5	0.97	0.009	Basp1	68.1	-1.43	0.001
H2-Oa	139.9	0.97	0.030	Myo1b	59.9	-1.42	0.017
Vax2	894.8	0.97	0.004	Armc2	53.2	-1.42	0.044
Ifitm10	202.0	0.97	0.021	Rtkn	119.6	-1.41	0.001
Slc2a9	750.1	0.97	0.000	Snora31	50.9	-1.41	0.003
Klf11	755.8	0.96	0.003	Gbp2b	391.9	-1.41	0.000
1500015A07Rik	172.9	0.96	0.009	Parvb	60.9	-1.41	0.022
Fam117a	1922.8	0.96	0.000	Nfat5	3334.9	-1.40	0.000
2010320M18Rik	132.7	0.95	0.002	Rgs9	53.7	-1.39	0.016
Ifngr1	27510.3	0.95	0.000	Plod2	157.2	-1.39	0.001
Klf13	17718.8	0.95	0.000	Acacb	56.1	-1.39	0.009
Mvb12b	1412.5	0.95	0.000	Eif4a2	10052.8	-1.39	0.000
9330133O14Rik	484.5	0.95	0.004	Gm4841	239.7	-1.38	0.000
Tnfrsf26	1382.5	0.95	0.000	Ces2c	278.7	-1.37	0.007
Gm7120	166.2	0.95	0.006	Sash1	68.1	-1.37	0.017
Arl4c	12558.1	0.94	0.000	Dgat2	66.8	-1.37	0.000
Gramd4	6518.8	0.94	0.000	Gbp8	2507.4	-1.36	0.000
Gpc1	4053.8	0.94	0.000	Ankrd23	259.8	-1.36	0.000
Rasgrp2	8737.9	0.94	0.033	Hist1h1b	57.7	-1.35	0.021
Cercam	304.6	0.94	0.001	Myadm	716.8	-1.35	0.000
Sh3bp5	1737.1	0.94	0.000	Tmc4	61.6	-1.35	0.024
Acss2	1118.2	0.93	0.000	Hmgn3	121.7	-1.35	0.021
Tcta	382.3	0.93	0.002	Cables1	276.0	-1.35	0.001
Slc48a1	3234.0	0.93	0.000	Rgs1	3103.1	-1.35	0.000
Shf	69.4	0.92	0.019	Slc2a6	278.4	-1.35	0.000
Atp1b3	24190.1	0.92	0.000	Akap5	63.0	-1.34	0.023
Slc25a20	2972.8	0.92	0.000	Uckl1os	51.8	-1.33	0.031
Klhl6	6126.6	0.92	0.000	A630023P12Rik	221.4	-1.33	0.000
Tgfbr3	311.9	0.92	0.014	Dock4	114.4	-1.33	0.002
Man1c1	214.5	0.92	0.004	Spire1	96.5	-1.33	0.007
Tpcn1	2630.0	0.92	0.000	Zfp467	445.6	-1.32	0.000
Plxnd1	3085.9	0.91	0.000	Plcb4	270.8	-1.32	0.000
Cd69	3719.7	0.91	0.000	Dkk3	63.4	-1.32	0.033
Prf1	2403.0	0.90	0.001	Neat1	3175.9	-1.31	0.002
Myl4	372.3	0.90	0.004	Mx2	94.2	-1.31	0.001
Gm2a	6624.0	0.90	0.000	Bicd1	111.3	-1.31	0.002
Pgm2	2419.0	0.89	0.000	Ttll3	201.8	-1.31	0.001

Supplemental Table 4. Genes identified to be differentially expressed (adjusted *p* value <0.05) between KO and WT CD4+ T-cells. Genes with positive Log2 fold change (log2FC) values are upwardly expressed in KO T-cells (top 200 genes). Genes with negative log2FC values are downwardly expressed in KO T-cells (top 200 genes).

Supplemental Table 5. Differentially expressed genes in KO vs WT CD8+ T-cells.

Gene	Base mean	Log2FC	*p*-adj	Gene	Base mean	Log2FC	*p*-adj
Scn2b	207.3	3.69	0.000	Hspa1a	2025.4	-7.20	0.000
Myl10	69.9	3.68	0.000	Hspa1b	1212.5	-6.78	0.000
Igsf23	190.3	3.66	0.000	Krt17	58.7	-5.23	0.009
Dnajc6	376.3	3.62	0.000	Fosb	684.4	-3.85	0.000
Cd4	28376.7	3.47	0.000	Miat	404.6	-3.40	0.000
Cd79b	955.4	3.22	0.000	Ier5l	58.2	-3.23	0.001
Pygm	719.0	3.07	0.002	Mrc2	123.0	-3.19	0.034
Atp1b1	118.9	2.95	0.000	Plbd1	207.4	-3.16	0.000
Ston1	199.9	2.83	0.002	Celf4	75.4	-3.09	0.047
Klrb1c	773.7	2.82	0.000	Rasd2	59.0	-3.04	0.000
Tmprss4	79.3	2.80	0.007	Insrr	98.9	-2.96	0.000
Abca1	291.3	2.79	0.005	Fos	1509.8	-2.95	0.000
Tmem176b	729.4	2.71	0.000	Gm4841	108.6	-2.92	0.000
Emilin1	358.8	2.69	0.000	Rab7b	50.0	-2.86	0.000
G0s2	181.3	2.59	0.000	Coch	83.2	-2.76	0.000
Cabp1	65.1	2.53	0.000	Dkk3	84.1	-2.74	0.000
Islr	81.4	2.48	0.001	Lmntd2	72.4	-2.72	0.000
Qrfp	754.0	2.47	0.000	Jun	1930.5	-2.68	0.000
Cnr2	140.7	2.45	0.004	Naip5	168.1	-2.67	0.000
Tmem176a	500.5	2.43	0.000	Snord22	100.7	-2.59	0.000
Gpnmb	704.8	2.42	0.000	Clgn	284.0	-2.59	0.000
Gzma	44263.5	2.41	0.023	Map2	143.3	-2.58	0.018
Lrp5	909.0	2.37	0.000	Ankle1	328.2	-2.51	0.000
Wfikkn2	2008.9	2.33	0.000	Ltk	159.6	-2.50	0.000
Ifitm3	2724.5	2.32	0.000	Anpep	52.0	-2.46	0.002
Aqp9	195.3	2.32	0.000	Pisd-ps1	1105.4	-2.40	0.000
Eng	100.7	2.29	0.002	Zan	234.0	-2.40	0.000
Ccr9	1137.5	2.27	0.022	Mir7058	115.7	-2.37	0.000
Sh3pxd2a	169.5	2.18	0.000	Aldh1a1	63.4	-2.37	0.045
Endou	106.2	2.17	0.016	Zfp692	371.3	-2.36	0.000
Pgam2	130.6	2.17	0.000	Glp1r	64.7	-2.35	0.000
Fam109b	224.9	2.17	0.000	Ceacam16	74.1	-2.33	0.030
St8sia1	351.8	2.10	0.000	Lrrk2	68.1	-2.29	0.048
5830418P13Rik	56.0	2.09	0.000	Cxcl9	128.0	-2.26	0.009
Txnip	24635.0	2.07	0.031	Rnf165	75.0	-2.24	0.001
Pik3ip1	2358.2	2.06	0.000	4933439C10Rik	76.1	-2.22	0.000
Ldhb	539.8	2.05	0.000	Sgpp2	74.2	-2.22	0.001
Fes	1750.6	1.99	0.000	Ppp1r15a	2442.5	-2.19	0.000
Trat1	373.5	1.99	0.032	Tvp23a	81.4	-2.14	0.001
Alpk2	95.3	1.98	0.003	Rasgef1a	115.4	-2.14	0.013

Tgfbr3	160.6	1.94	0.017
Fam212a	122.6	1.92	0.000
Try4	119.2	1.91	0.036
Aipl1	89.4	1.89	0.009
Il7r	7189.1	1.88	0.001
Myh10	314.0	1.84	0.005
Mrc1	166.5	1.83	0.036
Pls1	156.3	1.82	0.002
Tgfb3	339.4	1.79	0.000
Ifit3b	566.4	1.76	0.016
P2ry14	320.2	1.76	0.000
Fcer1g	788.5	1.76	0.000
Lair1	1105.2	1.73	0.000
Ccdc122	84.6	1.71	0.013
Ifit3	1005.3	1.66	0.025
Bcl2	9176.0	1.65	0.004
Klhl6	7217.8	1.61	0.000
N4bp2	1728.0	1.60	0.000
Dtx4	329.3	1.58	0.010
D930028M14Rik	144.9	1.55	0.038
Nqo1	127.3	1.53	0.036
Tnfrsf26	928.6	1.52	0.000
Wbscr27	108.4	1.50	0.049
2810001G20Rik	821.9	1.49	0.000
Pde2a	3662.8	1.49	0.010
Fam101b	759.3	1.48	0.027
Nebl	104.2	1.48	0.042
Sh3d19	107.0	1.48	0.002
Zfp580	155.7	1.47	0.040
Zbtb11os1	142.3	1.47	0.000
Trib3	107.5	1.45	0.022
Gm16845	144.5	1.45	0.000
Ldlrap1	1589.9	1.43	0.000
Gm19705	324.7	1.43	0.000
Eya2	446.1	1.40	0.000
Npas1	58.2	1.40	0.031
9030617O03Rik	462.5	1.39	0.003
Apobec2	1005.0	1.39	0.000
Palm	1663.6	1.38	0.000
Arl4c	11026.8	1.38	0.000
Sestd1	321.3	1.37	0.001

Dnase1l3	512.3	-2.13	0.000
Ccr6	106.5	-2.12	0.002
4930431P03Rik	79.3	-2.12	0.000
Mir155hg	92.9	-2.11	0.000
Lima1	56.3	-2.11	0.003
Mir8113	52.5	-2.11	0.001
Lmtk3	512.7	-2.10	0.000
Snora81	94.4	-2.09	0.000
Slc9a5	198.9	-2.09	0.000
Celf5	56.6	-2.09	0.004
Pisd-ps2	320.4	-2.07	0.000
A930013F10Rik	87.6	-2.05	0.012
Dusp1	1832.9	-2.05	0.000
Wdfy4	367.2	-2.03	0.007
Ciita	111.5	-2.02	0.004
Arnt2	332.5	-2.01	0.015
Gm996	73.9	-1.99	0.018
Tmprss6	65.4	-1.99	0.028
Csf2ra	111.4	-1.97	0.000
Rgs1	2361.8	-1.96	0.000
Neat1	4691.7	-1.95	0.000
Mir7115	84.1	-1.94	0.000
Apol10b	98.5	-1.93	0.000
Lilr4b	5241.0	-1.92	0.000
Kit	130.4	-1.92	0.002
Tmem198	133.5	-1.90	0.000
Insl3	101.6	-1.89	0.009
Cadm1	84.5	-1.89	0.013
H2-Aa	760.3	-1.89	0.002
Slc2a4rg-ps	1133.5	-1.88	0.000
Csf1	3522.0	-1.87	0.021
Lpl	80.9	-1.87	0.021
Sema6d	81.2	-1.85	0.013
Tnfsf13b	113.1	-1.84	0.011
Atat1	81.9	-1.84	0.000
Asb2	2050.1	-1.84	0.000
Rassf6	112.7	-1.83	0.009
Nr4a2	1719.7	-1.82	0.027
Trpm2	64.7	-1.82	0.014
Echdc2	126.1	-1.81	0.002
Bcl2l14	49.7	-1.81	0.003

Rras2	2644.4	1.37	0.001	Cldnd2	102.9	-1.80	0.000
Tns2	532.1	1.36	0.023	Snord104	54.8	-1.79	0.001
Acss2	1020.2	1.35	0.000	Snhg20	175.3	-1.79	0.000
Rcn3	1045.9	1.35	0.001	Ypel2	298.8	-1.78	0.000
Dtx1	6730.7	1.34	0.042	Plekhn1	123.3	-1.78	0.029
Idh2	1199.7	1.34	0.000	Syk	729.1	-1.78	0.006
Tanc1	612.8	1.33	0.000	Naip1	59.2	-1.78	0.033
Ifitm2	2264.4	1.32	0.001	Ociad2	270.0	-1.78	0.040
Ttyh3	5390.7	1.32	0.003	Aif1	96.5	-1.77	0.000
Bbc3	1394.2	1.32	0.008	Tert	113.6	-1.77	0.000
Igf2bp2	53.1	1.31	0.015	4-Sep	145.0	-1.76	0.006
Treml2	529.8	1.28	0.000	Tnfaip2	166.2	-1.75	0.001
Platr17	63.4	1.28	0.048	Rhbdl1	116.2	-1.75	0.000
Il20rb	239.2	1.28	0.006	Lilrb4a	3411.6	-1.74	0.000
Zc3h6	516.9	1.27	0.004	Gatm	148.7	-1.74	0.001
Plxdc2	229.5	1.27	0.036	B4galt4	77.2	-1.74	0.020
0610009L18Rik	63.3	1.26	0.016	Gabbr1	1264.1	-1.73	0.000
Fkbp5	20827.6	1.26	0.000	Npnt	636.4	-1.72	0.023
Slc14a1	958.3	1.26	0.000	4930579G18Rik	165.8	-1.72	0.021
Gm17745	609.9	1.26	0.000	Vill	81.0	-1.72	0.025
Cdnf	86.1	1.25	0.015	Adamts10	3210.4	-1.70	0.000
Nmnat2	169.8	1.25	0.018	Tbc1d9	74.7	-1.70	0.001
Abhd15	247.6	1.25	0.002	Clec7a	55.2	-1.69	0.033
Avpi1	178.7	1.24	0.000	H2-Eb1	854.8	-1.69	0.011
Npc1	3525.8	1.23	0.000	Ifng	5891.3	-1.69	0.000
Slc2a9	648.8	1.23	0.001	Prrt2	67.7	-1.68	0.002
Ssh2	5766.3	1.23	0.000	Lppr3	260.2	-1.68	0.000
Pdcd4	10562.8	1.23	0.001	Snora64	74.4	-1.67	0.004
Mvb12b	932.1	1.22	0.003	Trp73	351.5	-1.67	0.003
Lef1	4736.7	1.22	0.000	Snx32	192.2	-1.67	0.000
Akr1c13	278.9	1.22	0.000	Dennd6b	237.8	-1.66	0.009
F2rl1	1036.9	1.21	0.000	Acrbp	125.8	-1.66	0.000
Gpr146	1499.3	1.21	0.000	Gm4956	207.8	-1.65	0.035
Nrarp	596.4	1.20	0.010	Chrm4	312.8	-1.64	0.003
Ssc4d	130.0	1.19	0.006	Rsrp1	5243.8	-1.64	0.000
Zyg11b	4607.3	1.19	0.000	Vwa5a	333.7	-1.64	0.003
Zfp36l2	22231.1	1.19	0.008	Snord35a	59.1	-1.63	0.009
Gm4285	197.3	1.18	0.015	Gpr162	76.1	-1.62	0.043
S1pr1	9681.3	1.18	0.002	Eps8l1	302.9	-1.62	0.000
Snhg4	518.9	1.18	0.002	Slc43a1	151.1	-1.62	0.002
1700056E22Rik	127.7	1.17	0.010	Icam5	71.9	-1.62	0.001

Tcp11l2	1875.0	1.16	0.000	Gm3636	140.5	-1.61	0.002
Spata6	530.7	1.15	0.003	H2-Ab1	1037.3	-1.61	0.028
Lgals9	7043.8	1.15	0.000	Xcr1	351.1	-1.60	0.000
Rab3d	1356.6	1.14	0.000	Cdh24	316.9	-1.59	0.000
H2-Oa	150.0	1.14	0.008	Dnase1l2	56.1	-1.59	0.015
5730508B09Rik	480.0	1.13	0.000	Ttc16	98.1	-1.59	0.000
Itga2	447.2	1.13	0.007	Enpp2	184.1	-1.59	0.001
1700096K18Rik	132.1	1.12	0.037	Cdk14	73.9	-1.58	0.001
Ada	461.9	1.12	0.000	Rccd1	326.4	-1.58	0.000
Timp2	1353.4	1.12	0.000	Marcks	244.0	-1.58	0.000
Klf13	17065.2	1.12	0.000	Sirt4	175.8	-1.57	0.000
Klhl5	1296.9	1.12	0.000	Tppp3	73.7	-1.56	0.023
9330175E14Rik	180.2	1.12	0.023	Ttbk1	170.8	-1.56	0.034
Vmac	396.7	1.12	0.004	Degs2	269.9	-1.55	0.001
Itfg3	1012.0	1.11	0.000	Pawr	93.3	-1.55	0.007
Piwil2	229.6	1.11	0.049	Mpeg1	704.8	-1.55	0.000
2310039H08Rik	394.2	1.11	0.000	Smarcd3	53.3	-1.54	0.026
Cpt1a	9056.2	1.11	0.045	Eif4a2	9481.6	-1.54	0.000
Cyp2s1	141.1	1.10	0.044	Acacb	110.0	-1.54	0.008
Eef2k	241.2	1.10	0.015	Rtkn	100.6	-1.54	0.002
Slc43a2	2992.9	1.09	0.000	Zfp467	226.6	-1.53	0.026
Serpinb9b	526.9	1.08	0.012	Tmem86b	137.7	-1.53	0.000
Fam117a	1990.2	1.08	0.001	9430015G10Rik	561.3	-1.53	0.000
Gramd4	6568.3	1.08	0.000	Myadm	614.5	-1.52	0.000
Kbtbd11	15827.3	1.07	0.000	Ptpn5	288.7	-1.51	0.003
Ddit4	14612.6	1.07	0.000	Kifc2	122.5	-1.51	0.007
Sh3bp5	1082.4	1.07	0.025	Rab3il1	77.3	-1.51	0.025
Tmem38a	61.2	1.07	0.026	Mir17hg	74.9	-1.51	0.005
Egln3	355.1	1.07	0.019	Agrn	245.6	-1.51	0.000
1700025G04Rik	430.3	1.07	0.045	Cd200r1	545.7	-1.50	0.006
Klf11	951.6	1.07	0.029	Rassf4	90.2	-1.49	0.004
Mfsd4	997.0	1.06	0.001	Prmt2	360.5	-1.49	0.005
A930005H10Rik	444.7	1.06	0.004	Izumo4	322.9	-1.49	0.000
Cd6	19775.7	1.06	0.000	Fam193b	2229.9	-1.48	0.000
Rreb1	473.5	1.06	0.000	Tnfsf4	145.2	-1.48	0.000
Ndst1	3266.6	1.06	0.001	Dvl1	1211.9	-1.48	0.000
Card6	1573.7	1.06	0.021	2010016I18Rik	385.2	-1.47	0.006
Lrrc75b	247.0	1.06	0.005	Fam129c	110.2	-1.47	0.000
Elk4	3379.9	1.05	0.001	Sec14l2	65.0	-1.47	0.018
Amica1	2263.3	1.05	0.000	Mical2	386.4	-1.47	0.000
Nckap1	644.7	1.05	0.003	Plod2	236.5	-1.46	0.004

H2-DMa	1441.5	1.05	0.000	Mir3101	61.8	-1.46	0.026
Gm2a	5885.0	1.05	0.000	Rsad2	474.9	-1.46	0.006
Bach1	1151.6	1.05	0.000	Klf4	128.7	-1.44	0.016
Cxcr4	2538.2	1.05	0.000	Kndc1	73.6	-1.44	0.005
Oasl1	135.1	1.05	0.035	Mcoln3	148.4	-1.44	0.046
Mtus2	65.1	1.04	0.025	Phlda1	588.6	-1.44	0.000
Rnf144a	270.2	1.04	0.043	Klre1	153.8	-1.42	0.031
Rassf3	1493.7	1.04	0.000	Reep2	122.5	-1.42	0.011
Tomt	153.8	1.04	0.003	Rasgrp4	97.6	-1.41	0.005
Atp1b3	25602.0	1.03	0.000	Dqx1	121.8	-1.41	0.016
H1f0	3273.8	1.02	0.011	Proser3	133.3	-1.40	0.000
Cirbp	3663.3	1.01	0.027	Leng8	4609.2	-1.40	0.000
Fbxo17	201.1	1.01	0.023	Plxnb2	102.7	-1.40	0.001
Slc39a8	198.5	1.01	0.048	Card10	276.4	-1.39	0.002
Clybl	213.8	1.01	0.000	Slc37a2	1267.2	-1.39	0.000
Cpm	286.3	1.00	0.002	Shtn1	147.8	-1.38	0.000
Atp13a2	2308.5	1.00	0.000	Cav2	66.4	-1.38	0.018
Acp5	6846.6	1.00	0.000	Recql4	540.9	-1.38	0.000
Vax2	1179.4	1.00	0.034	5430416N02Rik	186.9	-1.37	0.000
Satb1	12741.6	1.00	0.000	Bik	72.7	-1.37	0.014
Zfp652	1074.2	1.00	0.002	Ptprv	630.5	-1.37	0.000
Tspan3	2615.5	0.99	0.000	2700097O09Rik	193.8	-1.37	0.000
Tmem71	5192.5	0.99	0.000	Wdr90	1350.1	-1.37	0.000
Tbc1d2	828.0	0.99	0.049	Utf1	131.4	-1.36	0.017
Mylip	1303.9	0.98	0.006	Fbxo48	172.3	-1.36	0.000
Fbxo32	512.4	0.98	0.002	Trpt1	92.7	-1.35	0.006
4933421O10Rik	139.3	0.97	0.001	A230050P20Rik	519.7	-1.34	0.000
H6pd	2073.1	0.97	0.000	Id3	373.0	-1.34	0.033
Dyx1c1	268.0	0.96	0.004	Rimkla	259.5	-1.34	0.000
Etv4	95.8	0.96	0.005	Nr4a1	1560.2	-1.34	0.000
Slc25a20	3075.2	0.96	0.001	Crem	675.4	-1.34	0.000
Pxylp1	1353.7	0.96	0.000	Gm20605	295.8	-1.33	0.015
Tpcn1	2419.9	0.96	0.000	Oplah	57.0	-1.33	0.016
Pink1	5227.3	0.96	0.000	Prkcz	370.6	-1.33	0.000
Frat1	251.5	0.95	0.019	4930503L19Rik	905.3	-1.33	0.000
Rab37	2277.7	0.95	0.017	Pkn3	154.2	-1.33	0.009
Pdk1	2177.0	0.94	0.000	Serf1	49.6	-1.32	0.041

Supplemental Table 5. Genes identified to be differentially expressed (adjusted *p* value <0.05) between KO and WT CD8+ T-cells. Genes with positive Log2 fold change (log2FC) values are upwardly expressed in KO T-cells (top 200 genes). Genes with negative log2FC values are downwardly expressed in KO T-cells (top 200 genes).

Supplemental Table 6. Published gene sets enriched for genes upwardly expressed in KO vs. WT CD4+ T-cells.

**MSigDB C7 GeneSet**	***p*-adj**	**FDR**	**NES**	**Genes**
GSE22886_NAIVE_CD4_TCELL_VS_48H_ACT_TH2_UP	0.0017	0.0043	1.8033	Add3, Bcl2, Eef2, Islr, Lsr, Ndst1, Pcif1, Plac8, Plxdc1, Ptp4a3, Rpl29, Rpl3, Rpl32, Rpl8, Samhd1, Slc48a1, Smpd1, Tbxa2r, Tns2
GSE43955_10H_VS_60H_ACT_CD4_TCELL_WITH_TGFB_IL6_UP	0.0186	0.0045	1.4780	Akt1, Atp1b3, Bscl2, Chmp6, Eif3k, Emb Eya2, Gorasp2, Hdgf, Hic1, Imp3, Ints9, Plxnd1
GSE11057_NAIVE_VS_CENT_MEMORY_CD4_TCELL_DN	0.0260	0.0074	1.4264	Adam19, Capns1, Chst12, Cotl1, Efhd2, Ezr, Klhl5, Lgals3, Rab27a, Slc6a6, Tigit, Ubl3
GSE13738_RESTING_VS_TCR_ACTIVATED_CD4_TCELL_DN	0.0052	0.0120	1.6202	Cmtm7, Efhd2, Gm2a, Gna15, Gramd4, Lrrc28, Mbd2, Ndufa8, Ndufb5, Ppp2r5a, Rftn1, Suox, Sytl3, Urm1, Zadh2
GSE45739_UNSTIM_VS_ACD3_ACD28_STIM_WT_CD4_TCELL_DN	0.0017	0.0158	1.8562	Acaa2, Adipor2, Atp1b3, Chst12, Efhd2, Gzma, Gzmb, Il2rb, Mfsd10, Mt1, Mvb12b, Npc1, Pecam1, Pigt, Pitpnc1, Ppm1a, Prf1, Pts, Rab27a, Rftn1, Srsf9, Tpst2
GSE22886_NAIVE_CD4_TCELL_VS_MEMORY_TCELL_DN	0.0180	0.0197	1.4729	Arl6ip5, Atp5a1, Fut8, Gpd1l, Grk6, Isoc1, Lrrc8d, Pdcd4, Slc39a8, Tcf12, Vps33a
GSE37301_HEMATOPOIETIC_STEM_CELL_VS_MULTIPOTENT_PROGENITOR_DN	0.0314	0.0215	1.4688	Adgrg5, Bach1, Efhd2, Mocs2, Nek9, Pkn1, Serinc3, Stk4, Ykt6
GSE37301_MULTIPOTENT_PROGENITOR_VS_COMMON_LYMPHOID_PROGENITOR_UP	0.0017	0.0002	1.8754	Ablim1, Acsf2, Add3, Cdk19, Daxx, Fam78a, Gabrr2, Gpr146, Gpr18, Itga6, Jak1, Klf13, Lef1, Llph, Mcur1, Ndufa6, Npc1, Pgam2, Pik3ip1, Prf1, Ptp4a3, Rasa3, S1pr1, Samhd1, Ssh2, Tcta, Traf6, Treml2
GSE37301_MULTIPOTENT_PROGENITOR_VS_CD4_TCELL_UP	0.0017	0.0037	1.8511	Ablim1, Arl4c, Atp1b1, Commd8, Dnmt3b, Emid1, Entpd5, Fam78a, Gpr18, Gramd3, Idh2, Il17ra, Il1rl2, Lair1, Lef1, Pdlim1, Pecam1, Satb1, Stk4, Tbxa2r, Tmem203, Tmem71
GSE22601_DOUBLE_NEGATIVE_VS_IMMATURE_CD4_SP_THYMOCYTE_UP	0.0083	0.0093	1.5708	Ppp1r9b, Pts, S1pr1, Sestd1, Sh3bp5, Sumo3, Sytl3, Tamm41, Tax1bp1, Tbc1d20, Tbcel, Tcf12, Tmed9, Tomm20, Tpst2, Unc119b, Vim, Xpa, Zyx

Supplemental Table 6. Gene set enrichment analyses (GSEA) were conducted using the MSigDB C7 Immunologic Signature database. Select immune-related gene sets overrepresented in genes upregulated in KO vs. WT CD4+ T-cells. P-adj = adjusted *p*-value, FDR = false discovery rate, NES = normalized enrichment score. Last column represents genes considered key drivers of the enrichment signal.

Supplemental Table 7. Published gene sets enriched for genes upwardly expressed in KO vs. WT CD8+ T-cells.

MSigDB C7 GeneSet	*p*-adj	FDR	NES	Genes
GSE9650_EFFECTOR_VS_EXHAUSTED_CD8_TCELL_UP	0.0036	<1.00E-5	2.3623	Acp5, Ak3, Anapc16, Arl4c, B4galt1, Bnip3l, Brap, Bscl2, Cdkn2d, Cib1, Commd7, Dap, Eif3l, Entpd4, Fam117a, Fez2, Gdap2, Glipr2, Gpc1, Hadhb, Hipk1, Hsd11b1, Il17ra, Itgb7, Lef1, Lgals9, Mtch1, Pkp3, Plac8, Psmd13, Satb1, Scp2, Smpd1, Twf2, Usp22
GSE9650_EFFECTOR_VS_MEMORY_CD8_TCELL_UP	0.0201	0.0846	1.4417	Ak3, Capns1, Cdkn2d, Dap, Galnt4, Gdap2, Lgals3, Lgals9, Msrb1, Ndrg1, Ppp2r5c, Psmd8, Rap1b
GSE9650_EXHAUSTED_VS_MEMORY_CD8_TCELL_DN	0.0036	<1.00E-5	2.4012	Ablim1, Acp5, Anapc16, Api5, Arl4c, Arl6ip5, Bcl2, Bnip3l, Cd44, Clp1, Entpd4, Eya2, Fam117a, Gm2a, Gpc1, Hadhb, Il17ra, Il7r, Itgb7, Kcnn4, Kctd10, Lef1, Map1lc3b, Pdk1, Pik3r1, Pitpnc1, Plac8, Poldip2, Psmd13, Rreb1, Satb1, Slc9a3r1, Slco3a1, St8sia1, Suclg1, Taf11, Tm9sf2, Tram1, Twf2
GSE9650_NAIVE_VS_EFF_CD8_TCELL_DN	0.0075	0.0064	1.5405	Bscl2, Capns1, Dap, Fgl2, Hsd11b1, Lgals3, Mbd2, Msrb1, Psmd8, Rnf19b, Stard10, Txndc5, Ykt6
GSE9650_NAIVE_VS_EXHAUSTED_CD8_TCELL_UP	0.0036	<1.00E-5	2.0304	Ablim1, Acp5, Anapc16, Api5, Arfgap2, B4galt1, Brap, Cndp2, Dap, Eng, Eya2, Gm2a, Gsn, Hadhb, Hsd11b1, Idh2, Il7r, Itgb7, Kcnn4, Map1lc3b, Mtch1, Plac8, Psmd13, Rtcb, S1pr4, Satb1, Slco3a1, Tbcel, Tmem50b, Twf2
GSE30962_ACUTE_VS_CHRONIC_LCMV_PRIMARY_INF_CD8_TCELL_UP	0.0036	<1.00E-5	2.3480	Acss2, Aqp9, Arl2bp, Arl4c, Arl6ip5, B4galt1, Bnip3l, Cox7a2l, Cpm, Crebrf, Fam117a, Fam78a, G0s2, Pdlim1, Ppp2r5a, Pxylp1, Rap1b, Selplg, Slc9a3r1, Slco3a1, St8sia1, Sun2, Tnfaip8l2, Tspan5, Txndc5, Wfikkn2, Zfp652
GSE30962_ACUTE_VS_CHRONIC_LCMV_SECONDARY_INF_CD8_TCELL_UP	0.0036	<1.00E-5	2.1165	Acss2, Aqp9, Arl4c, B4galt1, Cbr1, Crebrf, Gimap1, Hid1, Il7r, Lair1, Lef1, Mylip, Pitpnc1, Slco3a1, Ssh2, Tanc1, Tceanc2, Tnfaip8l2, Tprgl, Tspan5, Usp3, Wfikkn2, Zfp652
GOLDRATH_NAIVE_VS_MEMORY_CD8_TCELL_UP	0.0061	0.0142	1.6176	Acp5, Atp1a1, Bzw2, Cdkn2d, Dap, Ddit4, Eng, Ezr, Gsn, Gucd1, H2-Oa, Idh2, Klhdc2, Lef1, Lgals3bp, Ndrg1, Pdk1, Plac8, Ppp2r5a, Rmnd5a, Satb1, Sptbn1, St8sia1, Tbcel, Tmem50b
KAECH_DAY8_EFF_VS_DAY15_EFF_CD8_TCELL_UP	0.0036	4.00E-5	1.9911	Ak3, Ap3s2, Cib1, Cndp2, Dap, Eif3c, Eif3d, Emc3, F2rl3, Gyg, H13, Lgals3, Lgals3bp, Lgals9, Mbd2, Plac8, Ppp2r5c, Psmd8, Tnfaip8l1, Usp3
GSE22601_DOUBLE_POSITIVE_VS_CD8_SINGLE_POSITIVE_THYMOCYTE_UP	0.0036	<1.00E-5	2.0585	Atg13, Atp1a1, Bcl2, Cnp, Cpm, Crebrf, Degs1, Eif2ak1, Entpd5, Etnk1, F2rl1, Fbxo32, Fbxo33, Il17ra, Il4ra, Kbtbd11, Klf13, Lgals3bp, Mbd2, Pde2a, Pdlim1, Prkacb, Pten, S1pr4, Slc35g1, Ssh2, Sun2, Tor3a, Trpc4ap, Ttyh3, Zyg11b

Supplemental Table 7. Gene set enrichment analyses (GSEA) were conducted using the MSigDB C7 Immunologic Signature database. Select immune-related gene sets overrepresented in genes upregulated in KO vs. WT CD8+ T-cells. P-adj = adjusted *p*-value, FDR = false discovery rate, NES = normalized enrichment score. Last column represents genes considered key drivers of the enrichment signal.
